# Identification of Key Pathways and Genes in SARS-CoV-2 Infecting Human Intestines by Bioinformatics Analysis

**DOI:** 10.1007/s10528-021-10144-w

**Published:** 2021-11-17

**Authors:** Ji-Chun Chen, Tian-Ao Xie, Zhen-Zong Lin, Yi-Qing Li, Yu-Fei Xie, Zhong-Wei Li, Xu-Guang Guo

**Affiliations:** 1 Department of Clinical Medicine, The Third Clinical School of Guangzhou Medical University, Guangzhou, 510150 China; 2 Department of Clinical Laboratory Medicine, The Third Affiliated Hospital of Guangzhou Medical University, Guangzhou, 510150 China; 3 Key Laboratory for Major Obstetric Diseases of Guangdong Province, The Third Affiliated Hospital of Guangzhou Medical University, Guangzhou, 510150 China; 4 Key Laboratory of Reproduction and Genetics of Guangdong Higher Education Institutes, The Third Affiliated Hospital of Guangzhou Medical University, Guangzhou, 510150 China

**Keywords:** SARS-CoV-2, Bioinformatics

## Abstract

COVID-19 is a serious infectious disease that has recently swept the world, and research on its causative virus, SARS-CoV-2, remains insufficient. Therefore, this study uses bioinformatics analysis techniques to explore the human digestive tract diseases that may be caused by SARS-CoV-2 infection. The gene expression profile data set, numbered GSE149312, is from the Gene Expression Omnibus (GEO) database and is divided into a 24-h group and a 60-h group. R software is used to analyze and screen out differentially expressed genes (DEGs) and then gene ontology (GO) term and Kyoto Encyclopedia of Genes and Genomes (KEGG) pathway enrichment analyses are performed. In KEGG, the pathway of non-alcoholic fatty liver disease exists in both the 24-h group and 60-h group. STRING is used to establish a protein–protein interaction (PPI) network, and Cytoscape is then used to visualize the PPI and define the top 12 genes of the node as the hub genes. Through verification, nine statistically significant hub genes are identified: AKT1, TIMP1, NOTCH, CCNA2, RRM2, TTK, BUB1B, KIF20A, and PLK1. In conclusion, the results of this study can provide a certain direction and basis for follow-up studies of SARS-CoV-2 infection of the human digestive tract and provide new insights for the prevention and treatment of diseases caused by SARS-CoV-2.

**Supplementary Information:**

The online version contains supplementary material available at 10.1007/s10528-021-10144-w.

## Introduction

In December 2019, pneumonia caused by a new type of coronavirus appeared in the world. The World Health Organization (WHO) initially named it COVID-19 (coronavirus disease 2019). Since then, the Coronavirus Research Group of the International Commission on Virology has officially named the virus severe acute respiratory syndrome coronavirus type 2 (SARS-CoV-2) (Harapan et al. 2020).

SARS-CoV-2 is a positive-stranded RNA virus with an envelope. It is spherical under a transmission electron microscope, with a diameter ranging from 60 to 140 nm and a unique peak length of 8–12 nm. It belongs to the coronavirus 2b lineage (Zhu et al. 2020; He et al. 2020). Studies have shown that SARS-CoV-2 infects host cells through the combination of spike protein and angiotensin-converting enzyme II (ACE2). It spreads faster and is highly stable, but it is not very lethal (He et al. 2020).

SARS-CoV-2 can spread through the respiratory tract, saliva, contact, and excrement; the possibility of transmission through aerosols is also high (Chan et al. 2020). COVID-19 lurks in the human body for about 6.4 days on average and anyone is susceptible to infection (Wang et al. 2020). Currently, the clinical symptoms of COVID-19 are mainly fever (90% or even higher), cough (about 75%), and breathing difficulties (up to 50%) (Cipriano et al. 2020; Huang et al. 2020; Li et al. 2020). Some patients have symptoms of gastrointestinal diseases, such as nausea and vomiting. Studies have found that SARS-CoV-2 is present in the feces of COVID-19 patients, which suggest the possibility of SARS-CoV-2 infecting the human digestive tract and causing a series of diseases (Han et al. 2020). Furthermore, according to WHO statistics, as of July 27, 2020, there were a total of 15,785,641 cases of COVID-19 and 64,016 deaths worldwide. COVID-19 has become a communicable disease that seriously endangers global public health. Therefore, this study aims to provide new ideas and methods for clinically diagnosing, treating, and limiting the spread of SARS-CoV-2 by studying how it infects the human digestive tract.

Bioinformatics is an essentially interdisciplinary field that uses aspects of computer science, mathematics, and statistics to store, manage, analyze, and interpret biological data (Jin et al. 2020). This study uses bioinformatics analysis to identify and analyze the differential expression of organisms under different conditions and then uses gene ontology (GO) analysis and Kyoto Encyclopedia of Genes and Genomes (KEGG) analysis to obtain the biological significance of DEGs and establish a visual protein–protein interaction (PPI) network, finally leading to a conclusion (Ostaszewski et al. 2020).

## Methods and Materials

### Data Collection

We obtained the GSE149312 (Lamers et al. 2020) gene expression profile as microarray data from the GEO database (https://www.ncbi.nlm.nih.gov/geo/). In the original study, the authors treated intestinal organ samples with SARS-CoV or SARS-CoV-2 for 24 or 60 h, respectively. RNA was then extracted under dilation conditions (Exp) or differentiation conditions (DIF). We only took the SARS-COV-2 infection group and blank group from the original study, grouping them according to the infection time. In each group, there were 4 infected samples and 6 control samples.

### Differentially Expressed Genes (DEG) Screening

To obtain the DEGs between the experimental group and the control group as well as the corresponding volcano map and heat map, R software (version 4.0.1) and pheatmap package were used in this analysis, and *P* < 0.05 and | log2 fold change (FC) |>   1 were set as the threshold for judging DEGs. (Xie et al. 2019 Jul) DEGs were considered to have statistical significance within this critical value. The online tool, Venny (version 2.1.0; https://bioinfogp.cnb.csic.es/tools/venny/), was then used to make the Venn diagram of the DEGs shared by the 24-h group and 60-h group. All R scripts have been reported to the GitHub repository. (https://github.com/authentic-zz/mycode.git).

### The Function and Pathway Enrichment Analysis of DEGs

R language was used to conduct the GO analysis and KEGG pathway analysis to acquire the biological significance of DEGs. GO is a commonly used approach in bioinformatics analysis. Its function is to annotate genes and their products (Gene Ontology Consortium 2006; Liang et al. 2016). Methods to recognize gene function annotations include the biological process (BP), cell component (CC), and molecular function (MF) categories. KEGG is also a commonly used bioinformatics database for integrating and analyzing a large number of data sets obtained from high-throughput experimental technologies, such as genome sequencing (Liang et al. 2016; Kanehisa and Goto 2000). *P* < 0.05 was considered significant.

### Visualization of the Establishment and Module Selection of PPI Networks and the Identification of the Hub Genes

To evaluate the interrelationship between DEGs and build a visual protein interaction network, the STRING online database (version 11.0; http://string-db.org/) was used. First, the DEGs were entered into the STRING online database and the required minimum cross-evaluation was set to a medium confidence level > 0.4. Second, because the initial PPI obtained in STRING was complicated, Cytoscape was used to build a visual PPI relationship network. The plug-in cytoHubba in Cytoscape was used to analyze the core gene modules of the PPI network complex (the default parameters) and define the top 12 genes of the node as the hub genes.

### Verification of the Hub Genes

The 12 defined hub genes were uploaded to GraphPad Prism (version 8.0.2) in the order of high to low. Then GSE150728 was analyzed by bioinformatics, and the data obtained passed t test and non-parametric test and *P* < 0.05 was used as the standard to screen for statistically significant hub genes.

## Results

### The Identification of DEGs’

**Fig. 1:**
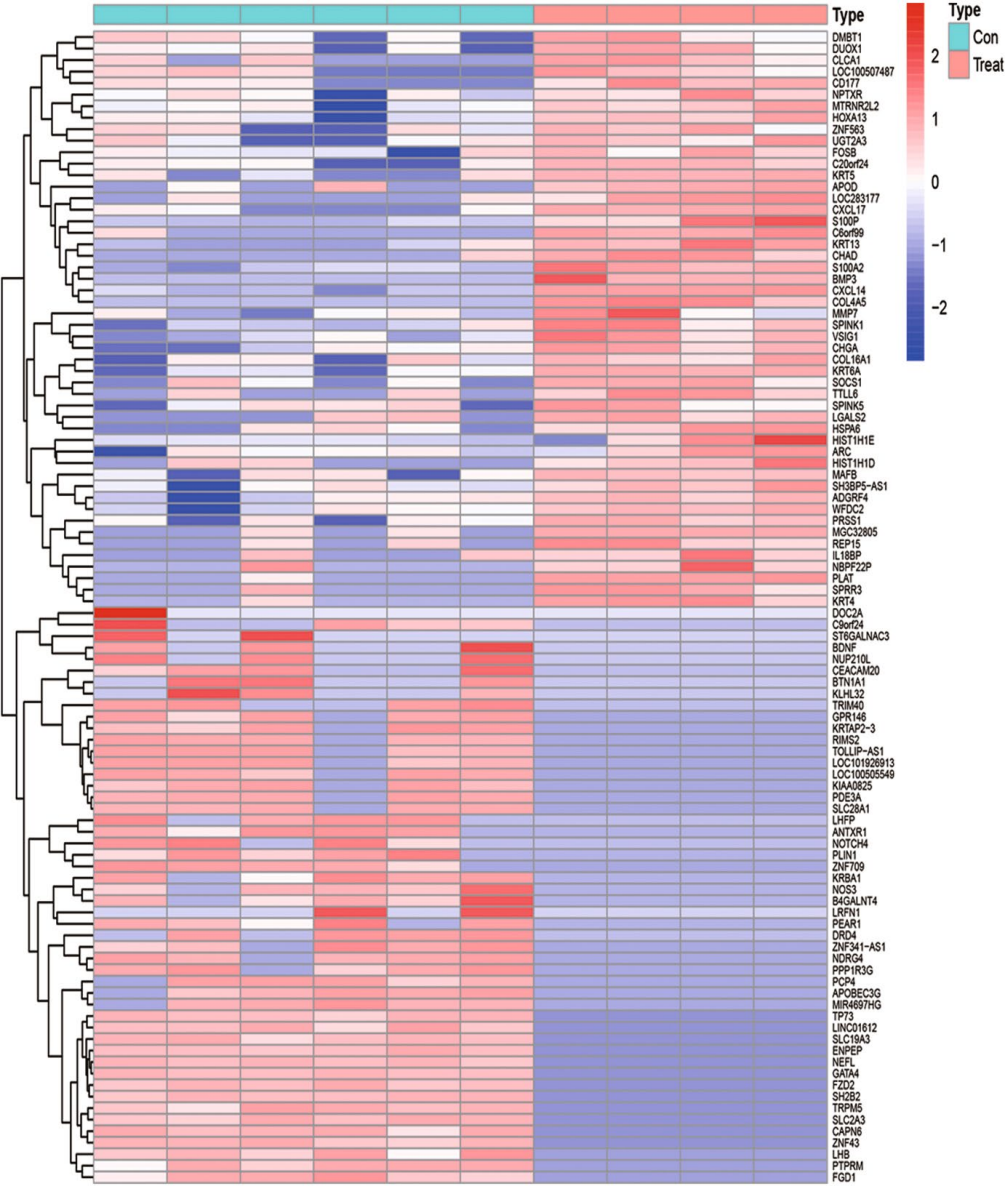
Heat map of the DEGS from the 24-h group

**Fig. 2:**
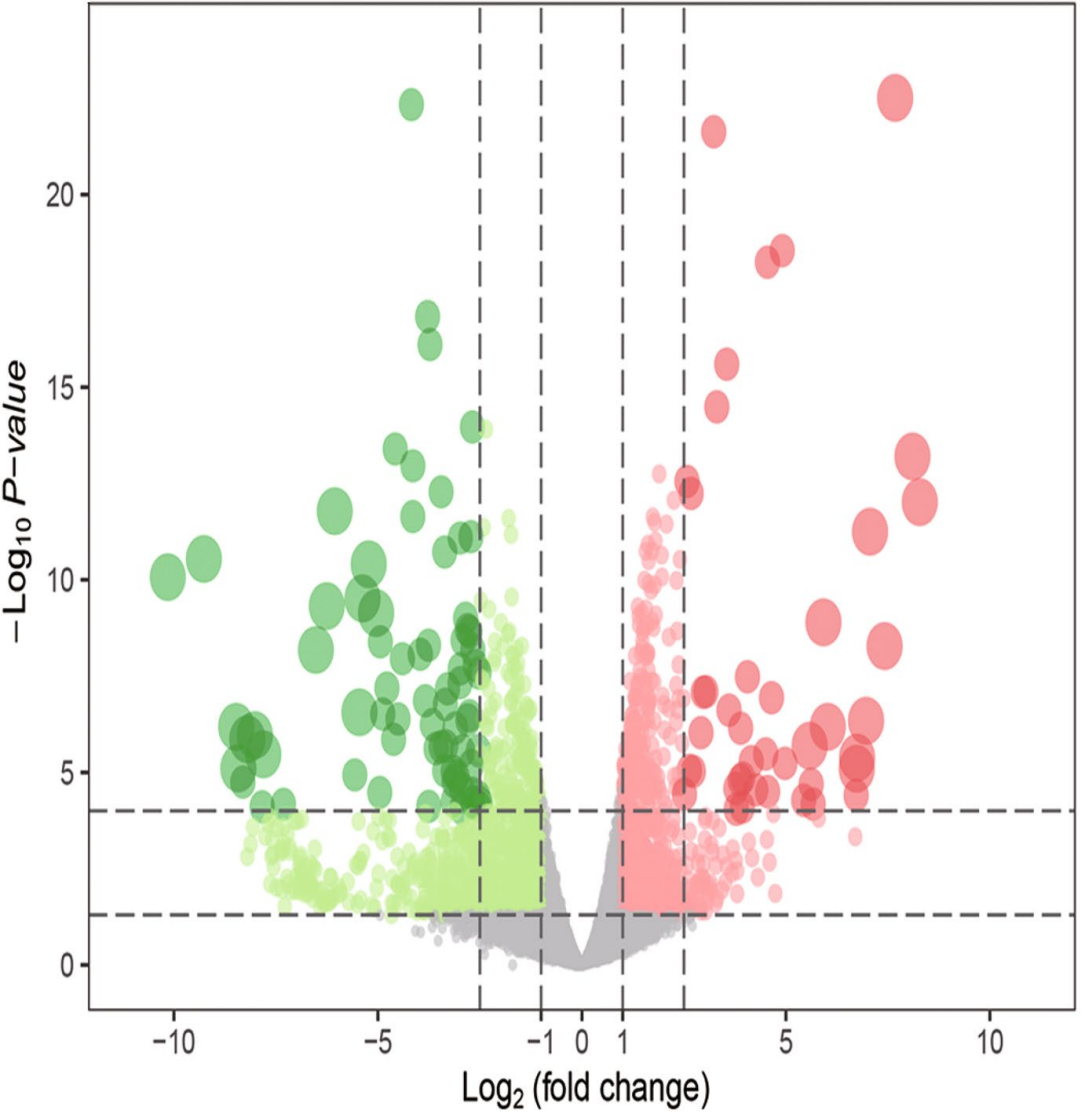
Volcano plot of the DEGS from the 24-h group

**Fig. 3:**
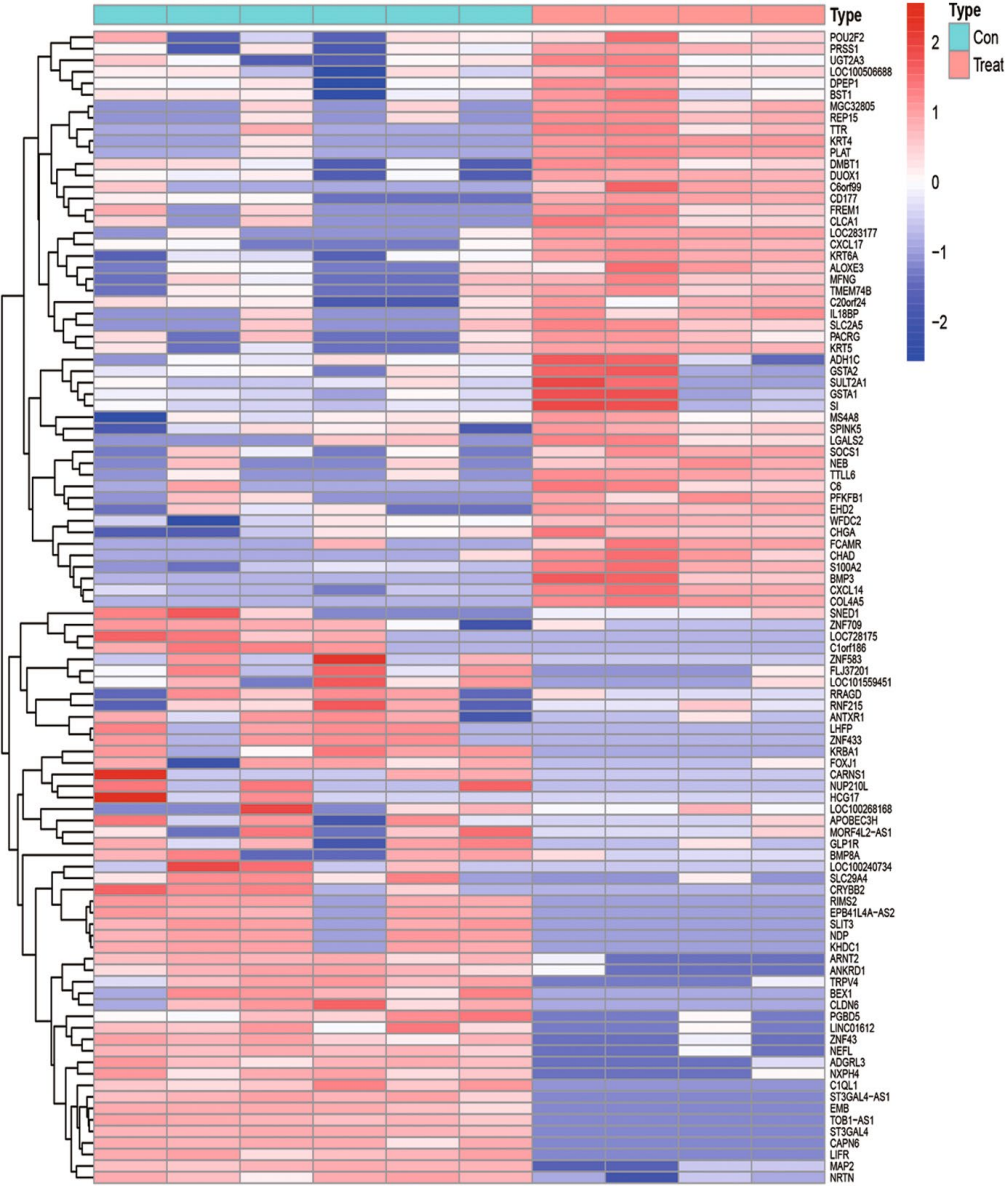
Heat map of the DEGS from the 60-h group

**Fig. 4:**
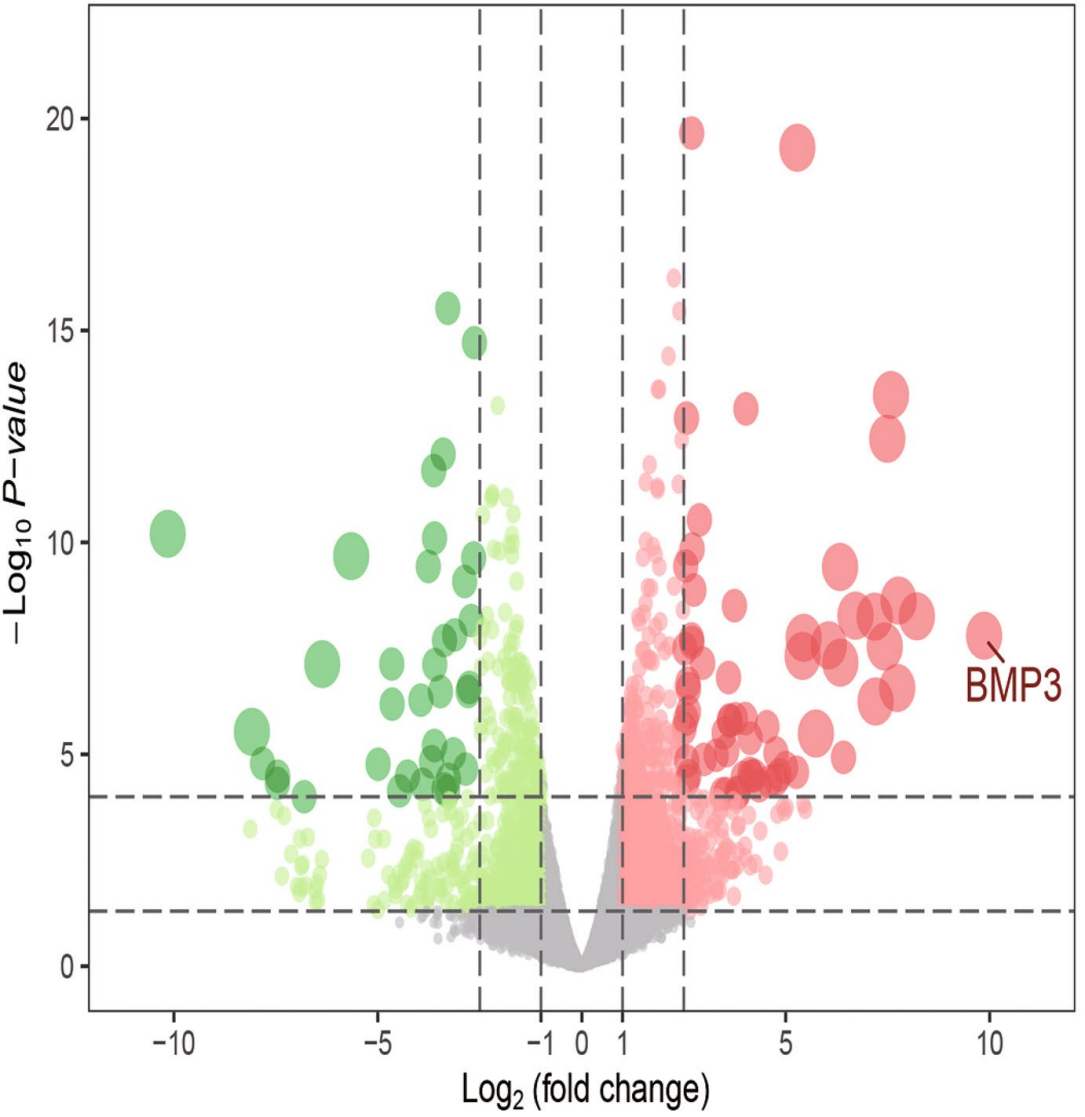
Volcano plot of the DEGS from the 60-h group

**Fig. 5:**
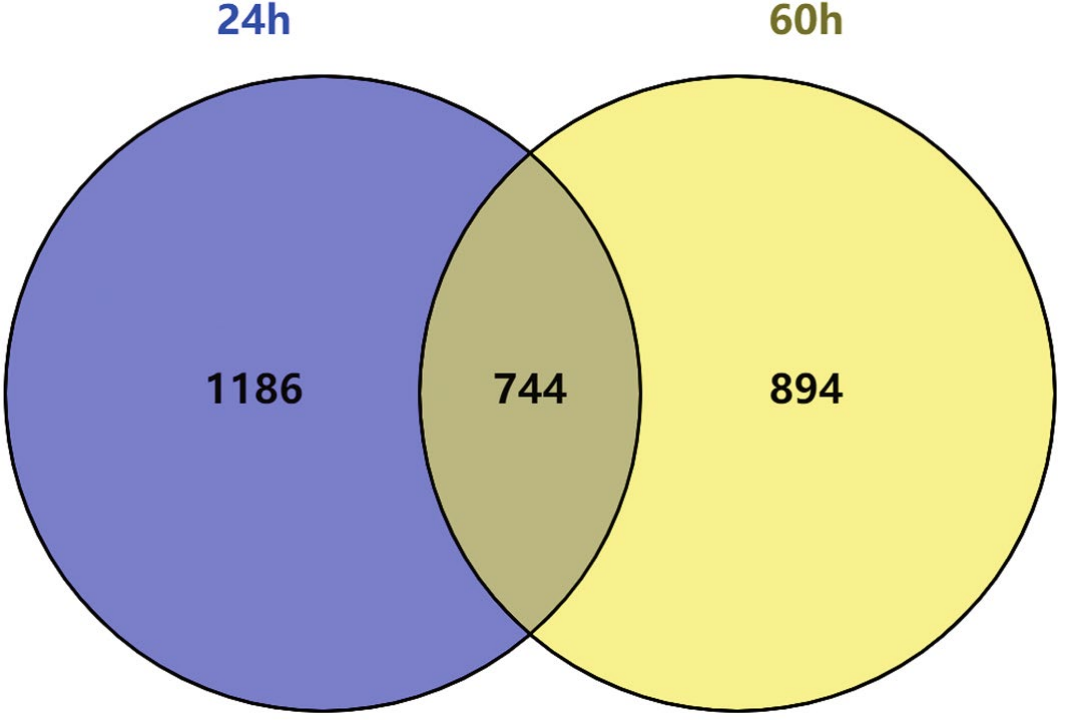
744 DEGs co-expressed between the 24-h group and the 60-h group

According to the established criteria, 1930 DEGs were screened from the 24-h group, including 748 up-regulated genes and 1182 down-regulated genes. These DEGs are shown in the heat map (Fig. 1) and a volcano plot (Fig. 2) of the 24-h group. From the 60-h group, 1638 DEGs were screened, including 890 up-regulated genes and 748 down-regulated genes. These DEGs are shown in the heat map (Fig. 3) and a volcano plot (Fig. 4) of the 60-h group. Through the analysis of the Venn diagram (Fig. 5), 744 DEGs co-expressed between the 24-h group and the 60-h group were obtained. More detailed data are shown in Table S1.

### GO Term Enrichment Analysis of DEGs

**Fig. 6:**
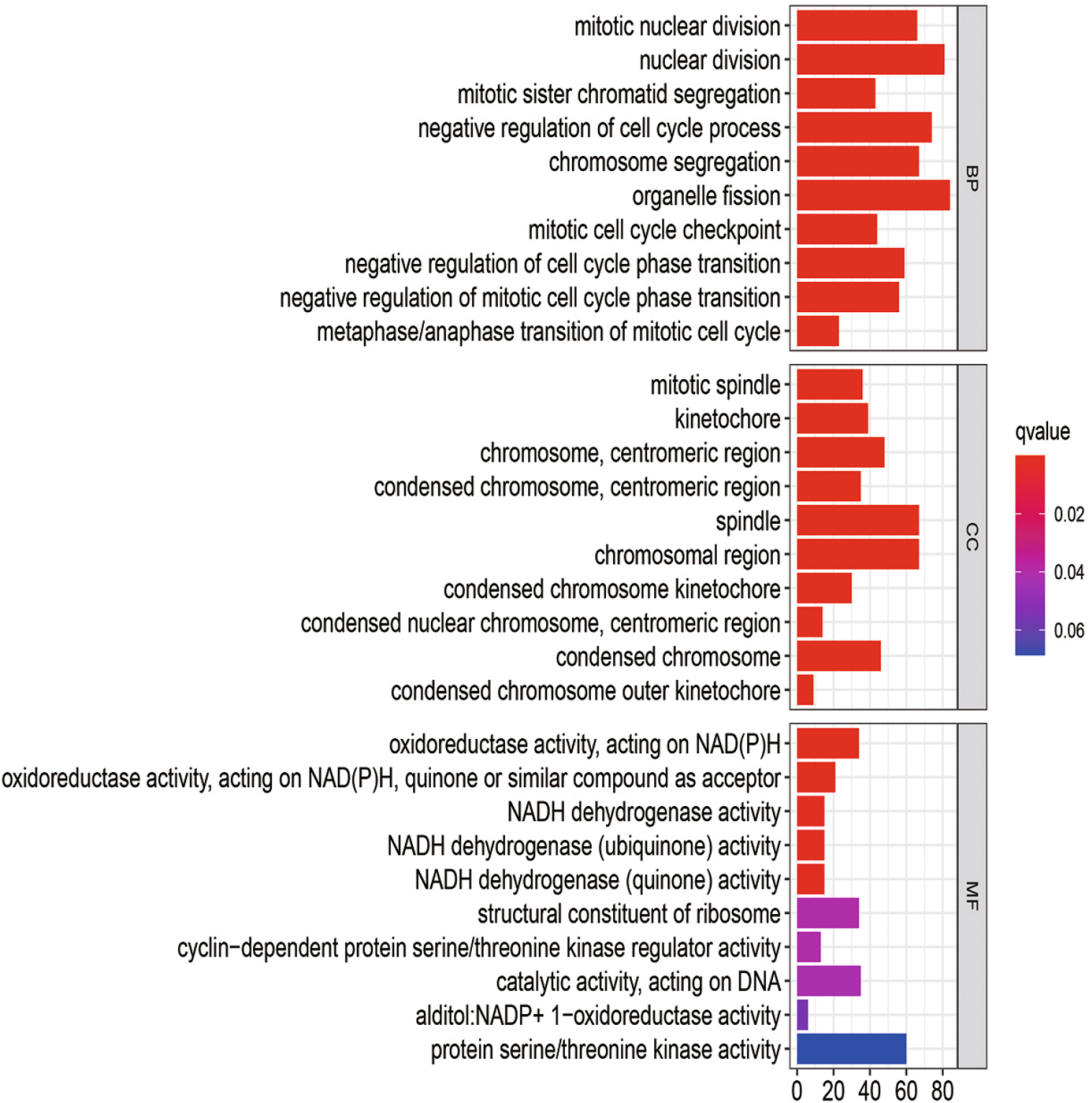
GO analysis of the DEGS from the 24-h group

**Fig. 7:**
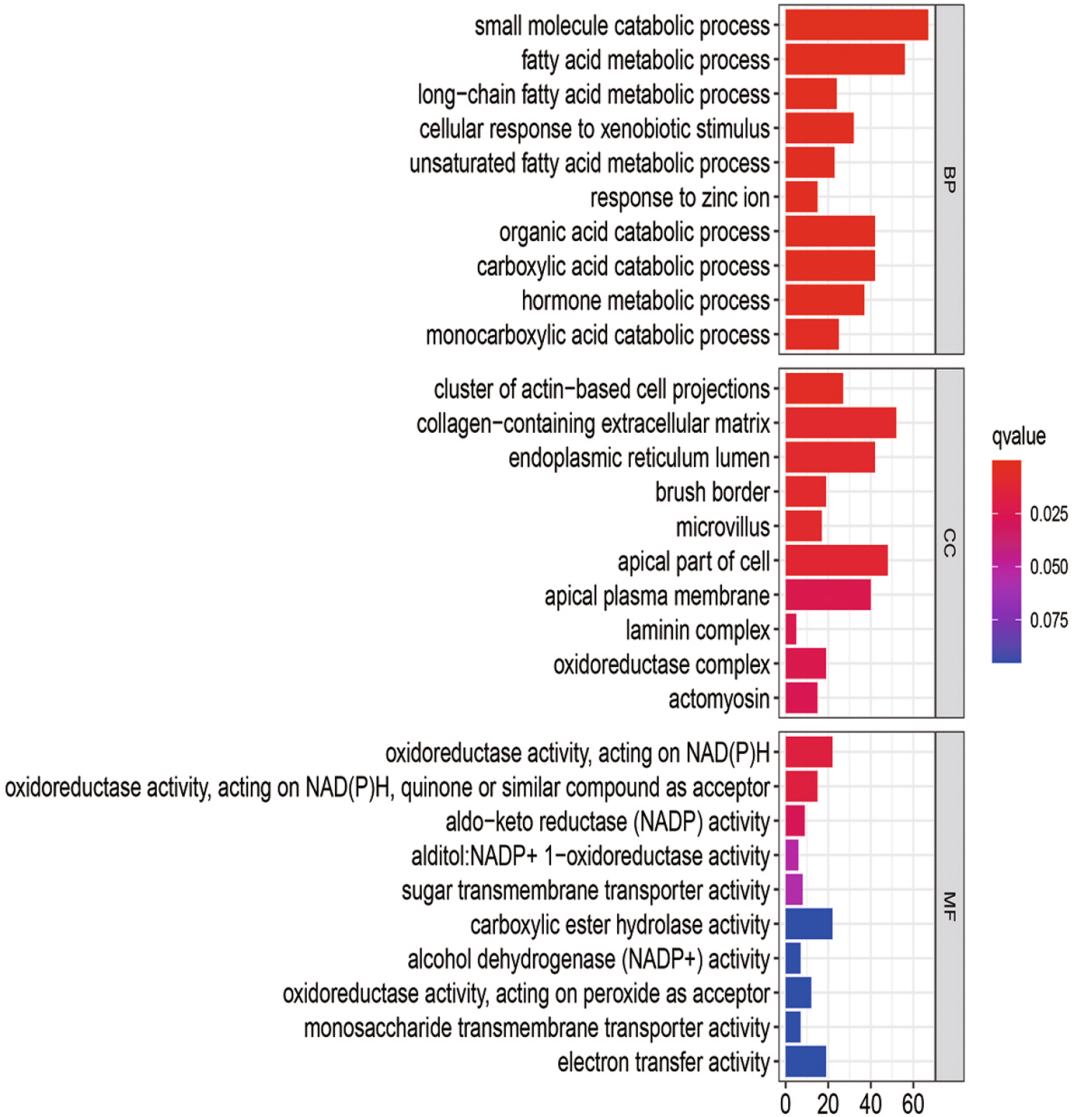
GO analysis of the DEGS from the 60-h group

The BP analysis showed that the DEGs of the 24-h group (Fig. 6) were significantly enriched in mitotic nuclear division, nuclear division, mitotic sister chromatid segregation, negative regulation of the cell cycle process, and chromosome segregation. The DEGs of the 60-h group (Fig. 7) were found in small molecule catabolic process and fatty acids and significantly enriched in acid metabolic process, long-chain fatty acid metabolic process, cellular response to xenobiotic stimulus, and unsaturated fatty acid metabolic process. Among them, there was no intersection between the 24-h group and the 60 h group. More detailed data are shown in Table S2.

### KEGG Pathway Analysis of DEGs

**Fig. 8:**
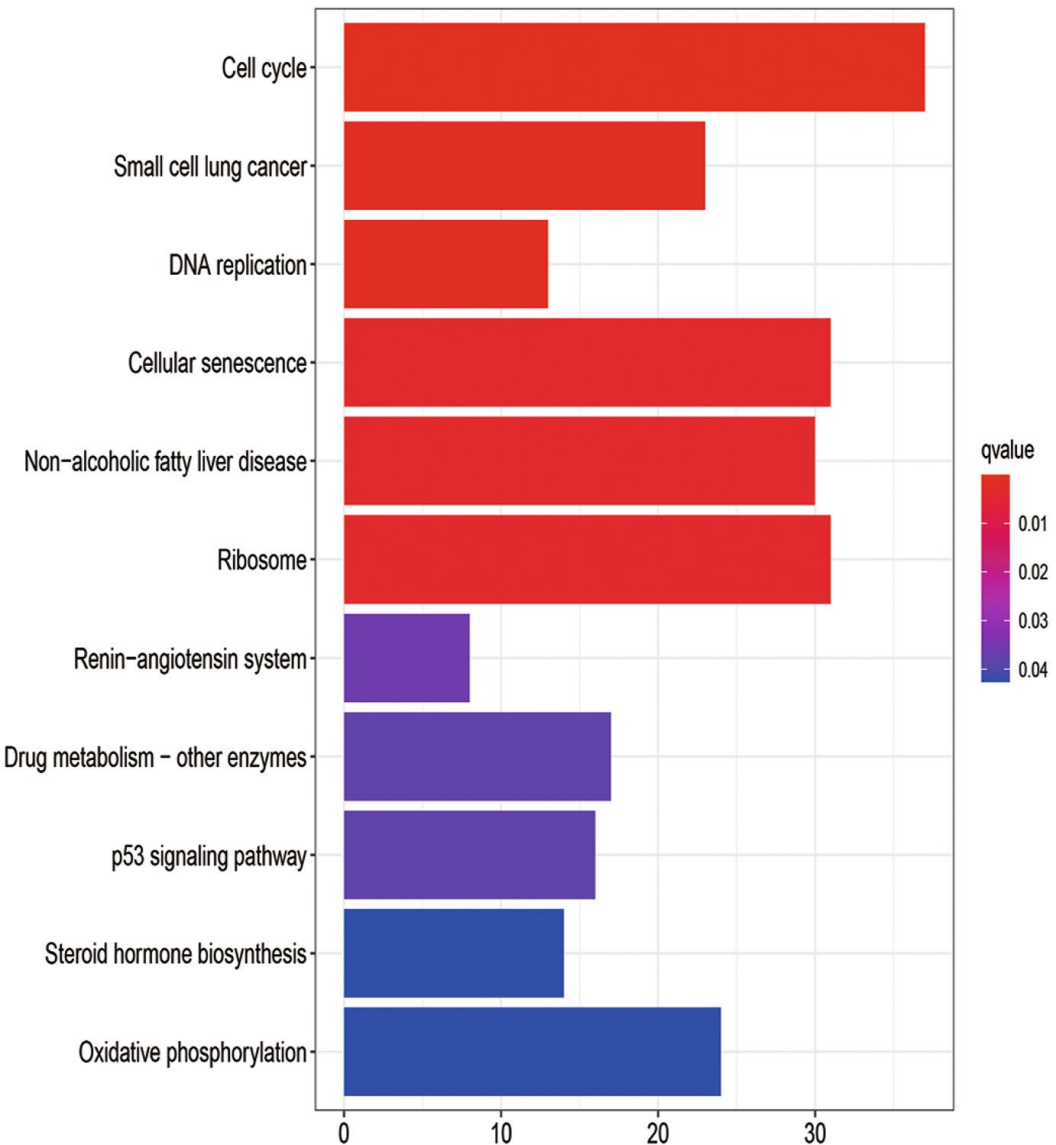
KEGG pathway enrichment analysis of DEGs from the 24-h group

**Fig. 9:**
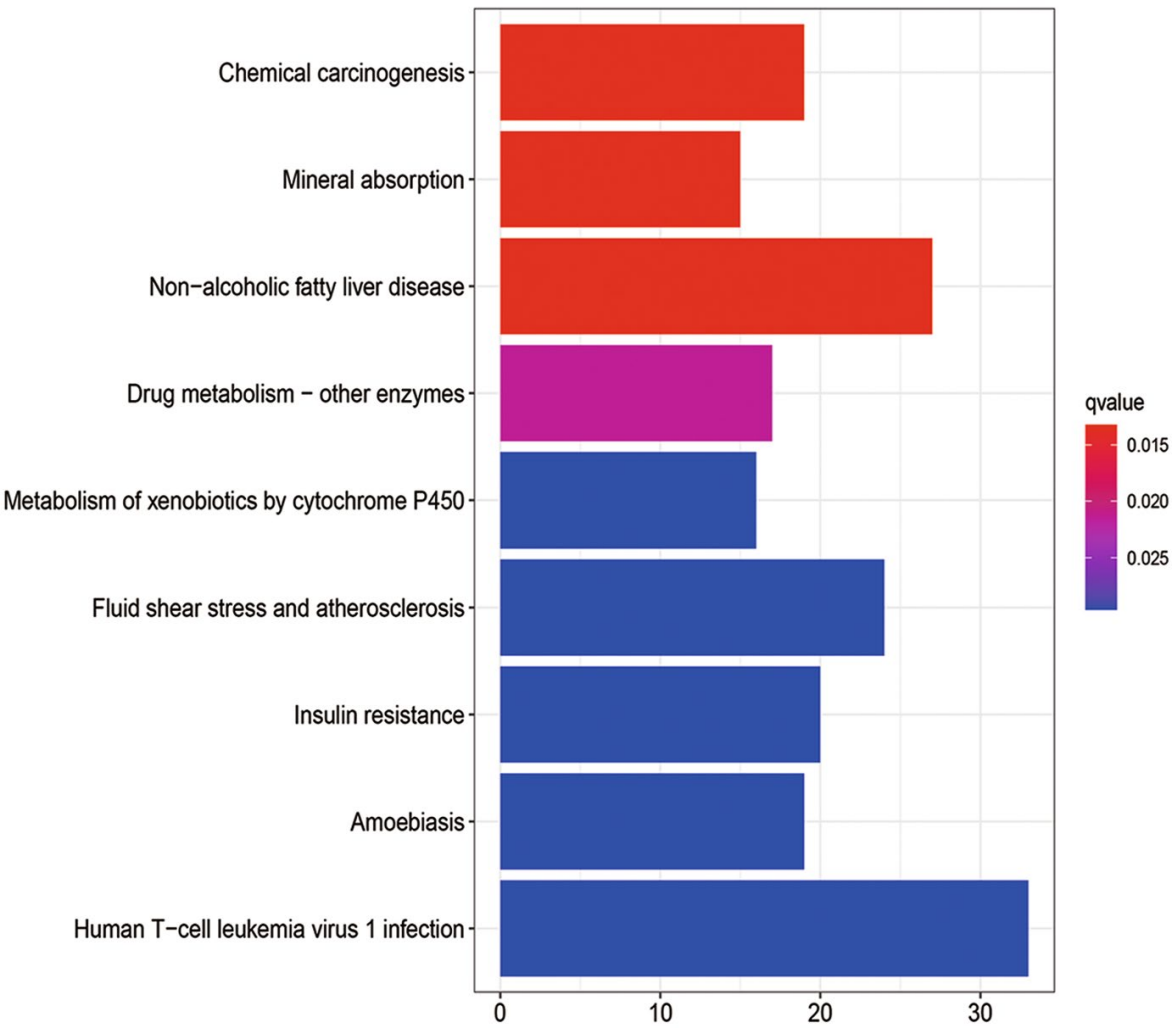
KEGG pathway enrichment analysis of DEGs from the 60-h group

In order to explore the potential mechanism of these DEGs, KEGG pathway analysis was performed using the R software. The results of KEGG analysis showed that the DEGs of 24-h group (Fig. 8) were significantly enriched in cell cycle, small cell lung cancer, DNA replication, cellular senescence, and non-alcoholic fatty liver disease. The DEGs of the 60-h group (Fig. 9) were found in chemical carcinogenesis and mineral absorption and significantly enriched in non-alcoholic fatty liver disease, drug metabolism-other enzymes, and metabolism of xenobiotics by cytochrome P450. Among them, non-alcoholic fatty liver disease was a pathway co-expressed in the 24-h group and 60-h group. More detailed data are shown in Table S3.

### Protein–Protein Interaction Network Analysis of DEGs

**Fig. 10:**
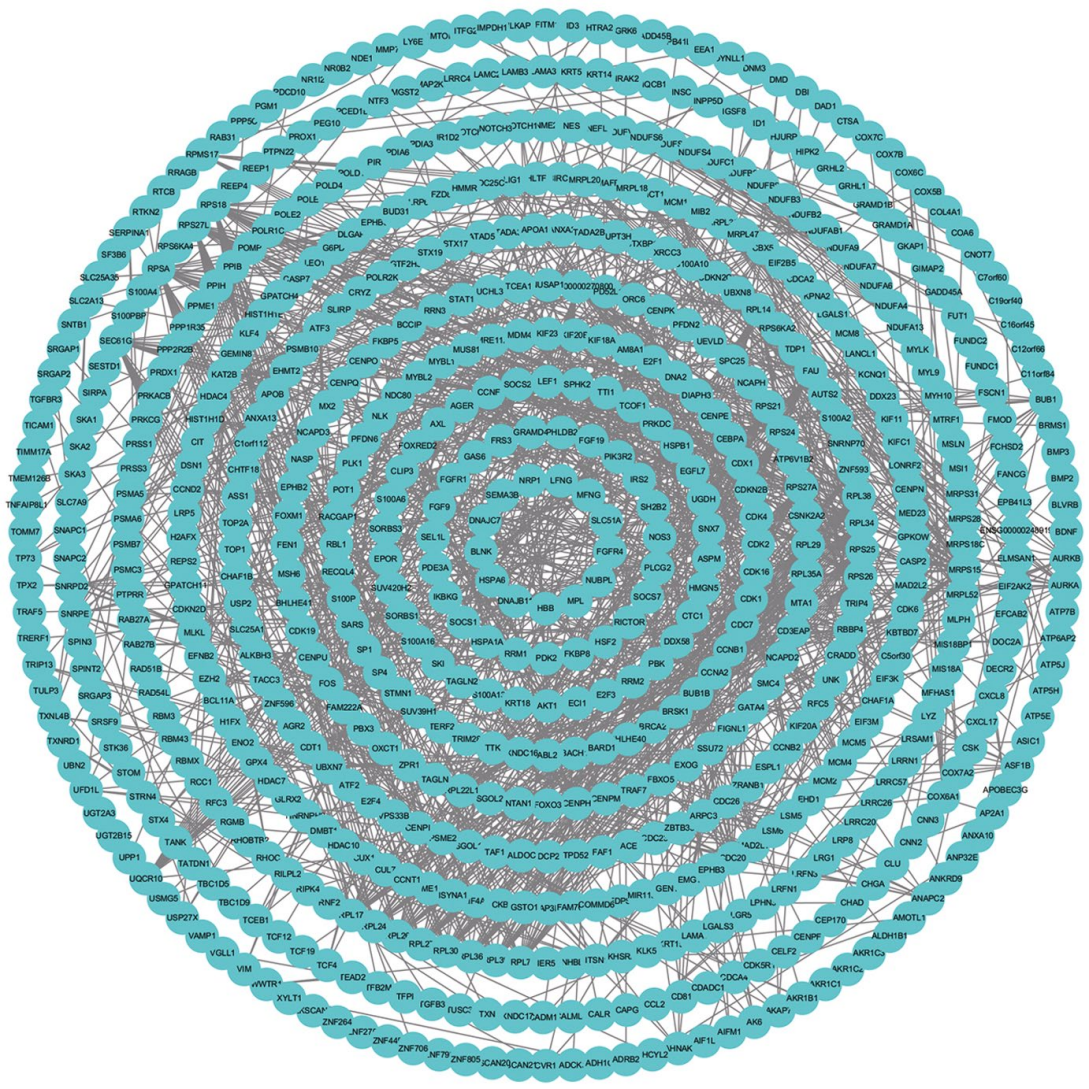
Protein–protein interaction (PPI) networks of the 24-h group

**Fig. 11:**
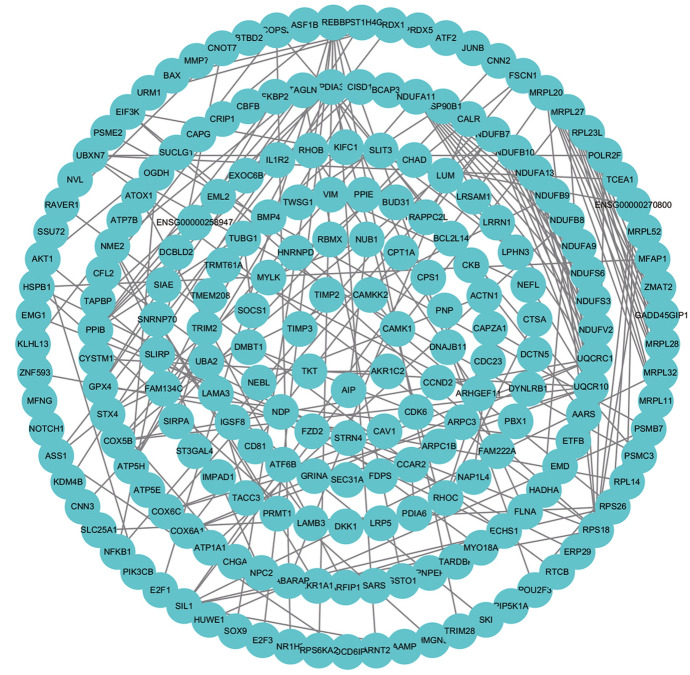
Protein–protein interaction (PPI) networks of the 60-h group

**Fig. 12:**
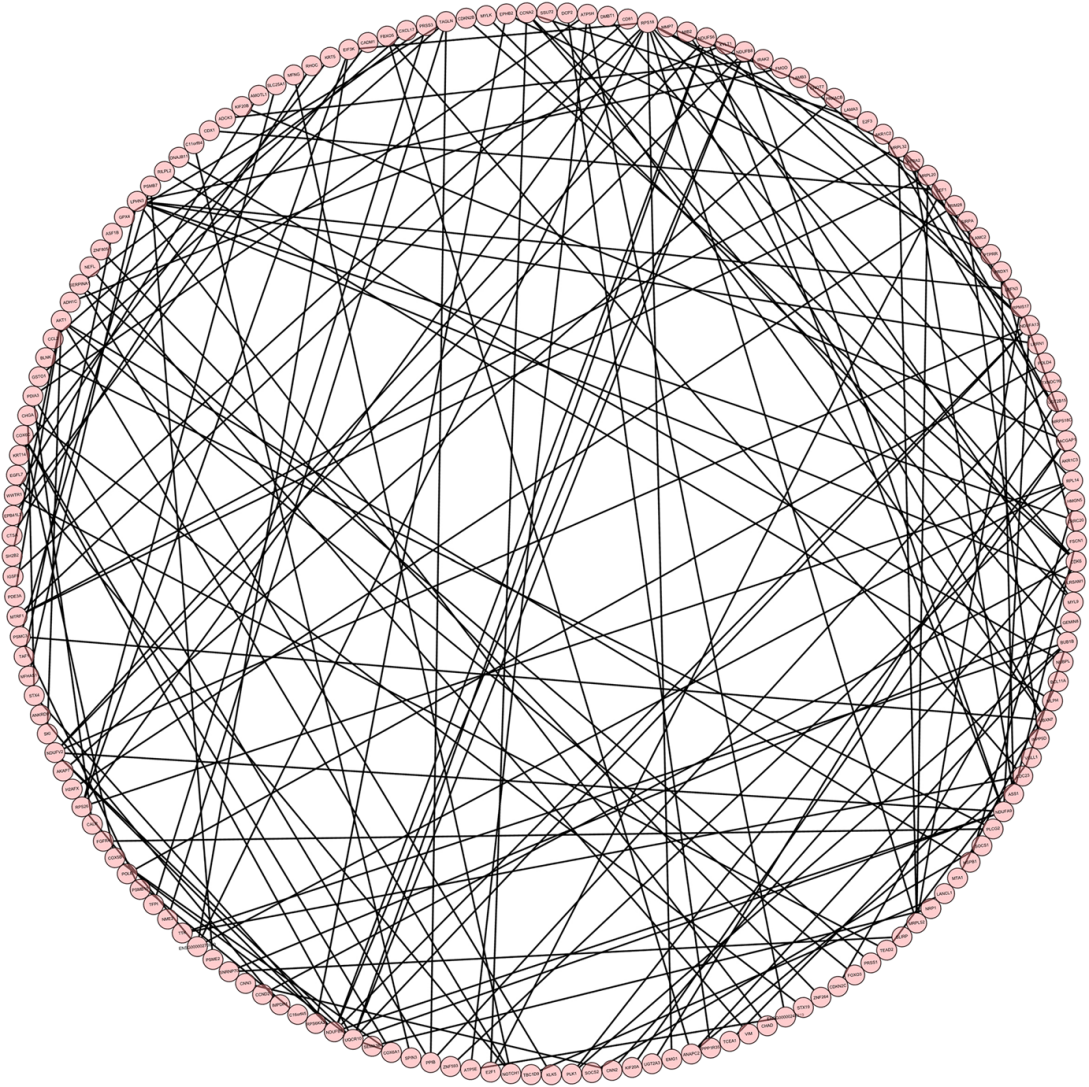
PPI network of the intersection between 24- and 60-h groups

**Fig. 13:**
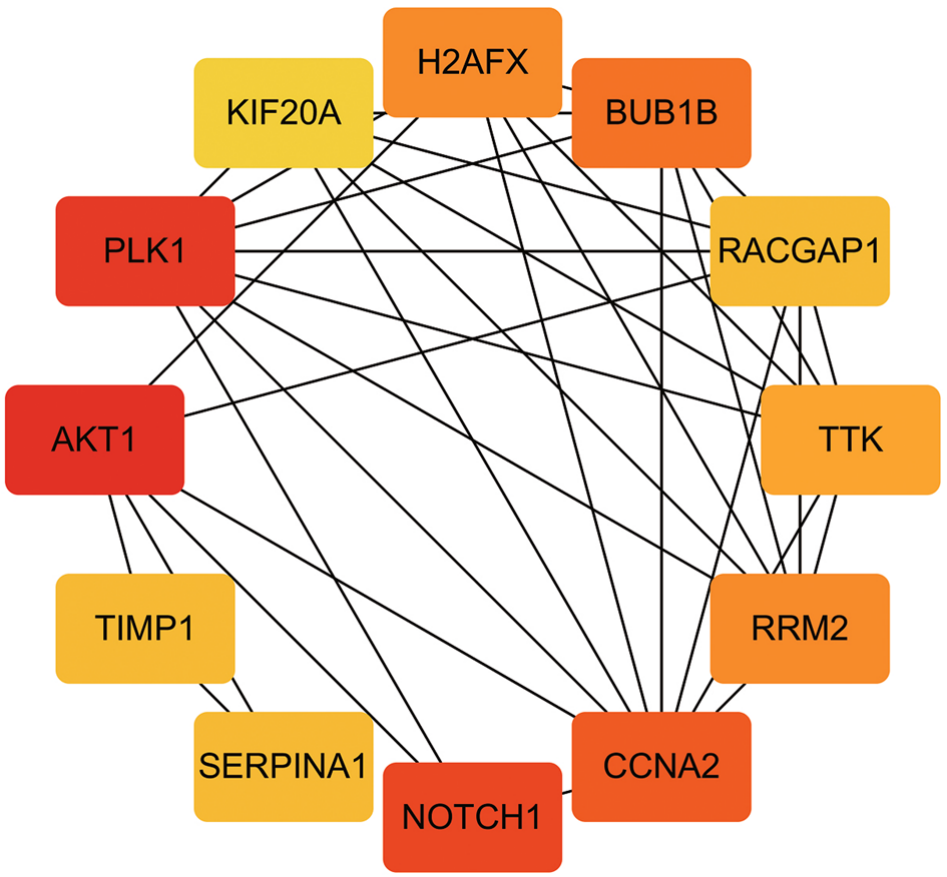
PPI network of the top twelve genes

Based on the STRING database, we constructed PPI networks of the DEGs in the 24-h group, 60-h group, and intersection group. The PPI network of the 24-h group (Fig. 10) included 1778 nodes and 1457 edges, the PPI network of the 60-h group (Fig. 11) included 710 nodes and 1457 edges, and the PPI network of the intersection group (Fig. 12) included 692 nodes and 162 edges. The top twelve genes (Fig. 13) were defined as the hub genes, namely AKT1, TIMP1, SERPINA1, NOTCH1, CCNA2, RRM2, TTK, RACGAP1, BUB1B, H2AFX, KIF20A, and PLK1.

### Verification of the Hub Genes

**Fig. 14:**
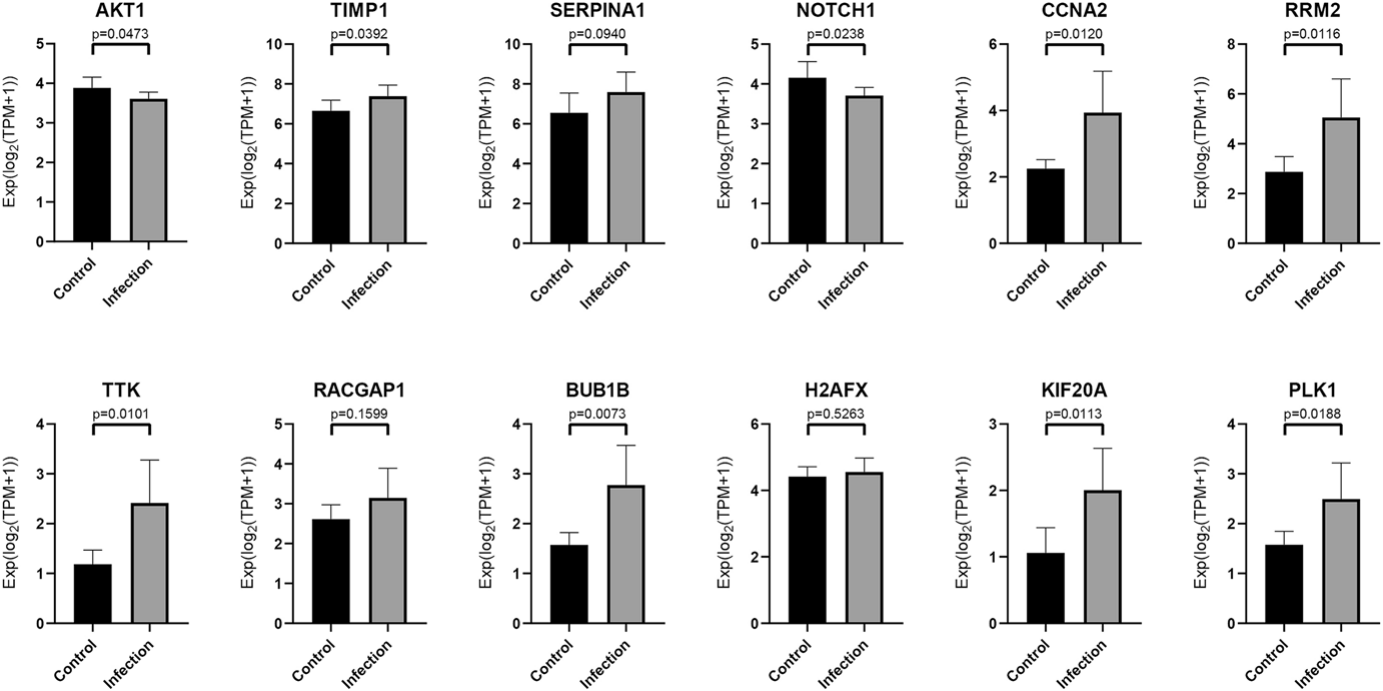
t tests and non-parametric tests of hub genes

Through *t* tests and non-parametric tests, with the standard set as *P* < 0.05, nine statistically significant genes (Fig. 14) were finally obtained, including AKT1, TIMP1, NOTCH1, CCNA2, RRM2, TTK, BUB1B, KIF20A, and PLK1. These genes may play a pivotal role in the impact of SARS-CoV-2 on the human intestine.

## Discussion

Currently, many types of mutations have appeared in SARS-CoV-2, but vaccination has not yet been popularized as vaccines are still in clinical trials. In this study, we identified the DEGs between the SARS-CoV-2 and the normal samples. To understand these DEGs better, we conducted GO function and KEGG pathway analysis on them and constructed a PPI network to determine the hub genes.

Angiotensin-converting enzyme (ACE2) is thought to be the mechanism of SARS‐CoV‐2-infected cells. SARS-CoV-2 activates the intestinal ACE2 receptor, causing inflammation (intestinal inflammation) and eventually diarrhea (Villapol 2020). Existing studies have shown that the high expression of ACE2 is not limited to lung type II alveolar cells (AT2); they are also found in the gastrointestinal tract, especially in the absorptive intestinal epithelial cells of the ileum and colon (Hajifathalian et al. 2020), which provide a scientific basis for the detection of SARS-CoV-2 RNA in the stool samples of some patients, indicating that the digestive system is a potential route of COVID-19 infection (Cipriano et al. 2020). Moreover, in the results of the BP process screening in the 60-h GO functional annotation group, we found that the cells' response to heterologous biological stimuli was significantly enriched. In previous studies, we found that following exposure to the initial stimulus, innate immune cells will experience metabolic, mitochondrial, and epigenetic reprogramming, leading to an immune response with an enhanced memory phenotype (Geller and Yan 2020).

In the analysis of the KEGG pathway, we found that the significant pathways in the 24-h group and 60-h group had a cross-path: non-alcoholic fatty liver disease. Intestinal dysfunction can cause changes in intestinal microbes and increase inflammatory cytokines, leading to the aggravation of symptoms and even more serious complications (Villapol 2020). An increase in the rate of abnormal liver function has been observed in patients with severe COVID-19. The microbiota can aggravate NAFLD through certain mechanisms, including changing the permeability of the intestines and the energy absorbed by the diet (Safari and Gérard 2019), so NAFLD patients may also be more susceptible to the increased cytokine production associated with COVID-19 (Prins and Olinga 2020). Studies have suggested that patients with NAFLD may be particularly vulnerable to SARS-CoV-2 infection and complications resulting from COVID-19 (Portincasa et al. 2020).

In GO analysis and KEGG pathway analysis, DEGs were found to be enriched in cell cycle. Coronavirus N protein is located in the cytoplasm and is involved in virus replication and assembly. Previous reports have indicated that the expression of the coronavirus N protein may affect the cell cycle (Zuwała et al. 2015). Both SARS and COVID-19 are considered to be pandemic infectious diseases caused by coronaviruses, which show that cell cycle research can play a significant role in their prevention and control.

Among the hub genes, the expression of TIMP1, CCNA2, RRM2, TTK, BUB1B, PLK1, and KIF20A were up-regulated, while the expression of ATK1 and NOTCH1 were down-regulated.

AKT1 is one of the subtypes of AKT. The down-regulation of AKT1 expression will promote the M1 polarization of macrophages, and M1 macrophages will secrete high levels of cytokines that can cause inflammation (Arranz et al. 2012). TIMP1 is a member of the tissue inhibitor of the metalloproteinase family (TIMP). As the coronavirus infection progresses, the expression of TIMP will increase correspondingly, and it is clearly expressed in lymphocytes, macrophages, and eosinophils during inflammation (Zhou et al. 2005). It can induce colon cell carcinogenesis through the FAK-PI3K/AKT and MAPK pathways (Song et al. 2016). The ATK1 and TIMP1 genes have a certain stimulating effect on immune cells, such as macrophages, and trigger the immune mechanism of the immune system. This indicates that SARS-CoV-2 may cause severe inflammatory bowel disease, fever, diarrhea, and other symptoms after infecting the human body, eventually causing damage to the human gastrointestinal system. Studies have shown that the overexpression of RRM2 and KIF20A usually accompanies the occurrence of cancer and plays a malignant role in cancer (Kitab et al. 2019; Morikawa et al. 2010; Sheng et al. 2018; Xiong et al. 2019).

In the results of this study, the down-regulation of NOTCH1 expression and the up-regulation of RRM2 and KIF20A provide a feasible direction for subsequent SARS-CoV-2 research. CCNA2 is the cyclin A2 gene, which is the gene encoding cyclin A2 on human chromosome 4 (Pagano et al. 1992). When a virus infects a cell, its genetic information can activate CCNA2 and promote the cell cycle. In addition, the overexpression of CCNA2 can enhance the reproduction, metastasis, and invasive ability of cancer cells and is closely connected to the occurrence and deterioration of ovarian cancer, liver cancer, and esophageal squamous cell carcinoma (ESCC) (Ruan et al. 2017). TTK, also known as MPS1, is a protein kinase. The up-regulation of TTK can activate PKCa/ERK1/2 to promote the division of colon cancer cells and the lack of TTK can lead to apoptosis (Zhang et al. 2017, 2019; Kaistha et al. 2014). The protein expressed by BUB1B is the BUB1 mitotic checkpoint serine/threonine kinase β, which plays an important part in the inspection of the spindle during mitosis. Furthermore, the expression of BUB1B in colon cancer tissues is higher than in normal colon tissues (Burum-Auensen et al. 2007). PLK1 is a member of the Polo-like family of mammals. It is located on the centrosome during mitosis and is usually overexpressed in cancer cells (Malumbres and Barbacid 2007). Moreover, PLK1 is also highly expressed in certain kinds of cancer, such as esophageal cancer and gastric cancer (Takahashi et al. 2003; Song et al. 2018). The TTK, BUB1B, PLK1, and CCNA2 genes and their translation products all perform significant tasks in the normal cell division cycle. During SARS-CoV-2 infection, the up-regulation of these four genes not only provides favorable conditions for the spread of the virus but also hints at the possibility of chromosomal abnormalities and other genetic material damage in the host cells. This result corresponds to the aforementioned expression results of NOTCH1, RRM2, and KIF20A. Therefore, the results of this study can provide a certain direction and basis for subsequent researchers for exploring the relationship between SARS-CoV-2 and cancer.

In conclusion, the nine hub genes obtained through statistical tests in this experiment all play an indispensable role in cell growth, reproduction, and disease. Therefore, this study analyzed their differential expression during the SARS-CoV-2 infection process, aiming to understand the subsequent diseases that SARS-CoV-2 may induce so as to facilitate their timely prevention. However, this study has certain limitations. For instance, the sample size of the experimental data could be further expanded, and the intestinal organs could not perfectly simulate the human environment.

## Supplementary Information

Below is the link to the electronic supplementary material.

Supplementary file1 (DOCX 34 kb)
Table S1: List of DGEs considering P < 0.05 and | log2 fold change (FC) | > 1

Supplementary file2 (DOCX 17 kb)
Table S2: Significantly enriched GO terms of DEGs

Supplementary file3 (DOCX 16 kb)
Table S3: Significantly enriched KEGG terms of DEGs
