# Supplementary material for: Identification of Key Pathways and Genes in SARS-CoV-2 Infecting Human Intestines by Bioinformatics Analysis
Source: Biochem Genet. 2021 Nov 17;60(3):1076–94. doi: 10.1007/s10528-021-10144-w (PMC8596852; doi:10.1007/s10528-021-10144-w)
Supplement: Supplementary file 1 — Supplementary file1 (DOCX 34 kb) Table S1: List of DGEs considering P < 0.05 and | log2 fold change (FC) | > 1 [file 10528_2021_10144_MOESM1_ESM.docx]

| 24h | DEGs | Gene terms |
| --- | --- | --- |
|  | Up | PLAT, CHGA, KRT6A, C6orf99, CXCL17, LOC283177, KRT4, HIST1H1D, COL4A5, BMP3, HSPA6, KRT5, REP15, CHAD, CLCA1, MGC32805, CD177, ARC, CXCL14, NBPF22P, DUOX1, HOXA13, TTLL6, PRSS1, S100A2, SPINK5, C20orf24, SPRR3, SH3BP5-AS1, SOCS1, WFDC2, UGT2A3, SPINK1, MAFB, HIST1H1E, VSIG1, ADGRF4, DMBT1, IL18BP, MTRNR2L2, MMP7, COL16A1, NPTXR, LGALS2, KRT13, ZNF563, S100P, APOD, FOSB, LOC100507487, ADH1C, ZNF213-AS1, ANO1, ATP5J2-PTCD1, FCAMR, IL1R2, SLC38A5, LOC105371049, OR7E91P, MST1L, SNORA63, RGP1, HERC2P9, EHD2, RPS10-NUDT3, ZNF470, CCR6, RN7SK, LOC100506746, LOC102724699, ANO7, ANXA13, PTPN22, CRYBG3, LEF1, ELMSAN1, DISC1-IT1, DNM3, NRCAM, MFNG, C6orf52, VSTM2L, LINC00630, RAD51B, PTAFR, PROC, HBEGF, NR0B2, FZD8, CCDC17, RGS2, CCK, PSORS1C2, ZNF623, VAMP5, C11orf98, GLB1L, IGFL1, AREG, LOC105377924, PAOX, RGCC, ENPP7P13, IRAK2, MRPS18C, ACPP, SIRPA, AKR1C2, SNORA61, CYP27B1, ALOX5, PRSS3, LINC00670, LYPD6B, LINC00483, LINC00506, LAMC2, TRIM29, RAB12, ZG16, LOC101927391, ASS1, NME2, BCAS1, LAMA3, XYLT1, ZNF222, KRT15, NR4A2, LY6D, OSBP2, SLC7A8, INSC, UBAC2-AS1, C8orf4, TTC39A, AHNAK2, BST1, CAMK1D, RPS26, PID1, ZBTB11-AS1, MTG2, GADD45G, EGFL7, FGF19, NMB, FAXDC2, MS4A8, CBR3-AS1, ZNF575, KIAA1549L, GPANK1, LOC101929415, HLA-F-AS1, CYP2E1, PPIB, SMIM5, DQX1, INCA1, FGFBP1, HOPX, SNORD104, LOC101928881, ACKR4, CLIC6, LONRF2, MINK1, LGALS1, HSPB1, RBP4, LOC102724094, CLCF1, RAB27A, CDX1, ACR, ATAD3B, RPS14P3, LOC102724908, RAB3B, SEMA7A, LOC101928936, TMEM265, SEMA6A, STOM, FCGBP, ZNF593, SOSTDC1, OR2A4, COX16, BLVRB, INPP5D, PHACTR3, AMPD3, CCL2, CAPN9, MT-TV, RPS6KA4, AP2A1, NTAN1, CARD6, MRPS31, C15orf48, ANXA10, LOC115110, AXDND1, ESAM, CCND2, OR7E14P, LOC100506023, 43893, LOC652276, CCDC144B, SLC2A6, UPP1, GRIP1, NRP1, PPP1R36, KCTD1, SLC44A5, EXOG, TXNDC17, PRELID1, PSMB7, SLIRP, BLOC1S1-RDH5, BHLHE41, LENG8, CCDC88B, NACA, CXCL1, C19orf70, UQCRBP1, KRT19, ZNF101, DYRK1B, LOC101928103, MIR4435-2HG, PDZK1IP1, CAPN8, ATP7B, GPR85, CRADD, TM4SF1, EREG, TIMP1, SULT1C3, MTRF1, AGR3, PCAT6, SPON1, AIMP2, ZNF430, FUT6, GCSHP3, ROR1-AS1, TXN, BCL2L2, GJB5, ICAM3, FAM76A, PGM1, ECM1, ZFP30, FAM83F, MRPL32, ATP9B, GTF2IRD2, EPHA1, PLCG2, DPCR1, CD68, GOLIM4, CYP2C18, COL4A1, HSPA1A, PRKACB, ARPC3, TMEM126B, PFDN6, SLC45A3, COA6, RPL24, LGALS4, C19orf33, FAM219A, TRIM7, SMIM12, RPS18P9, MRPS36, LMO4, GLRX2, STX19, MRPL20, RPS18, GPATCH4, LGALS3, NTPCR, SHFM1, AKR1B10, HTRA2, RAB31, MT-TC, BTNL3, RPS27A, PEAK1, SNHG12, NDUFB3, S100A6, ILKAP, UPF3B, LAMB3, PLCL2, PSMC3, STC2, TXNL4B, USMG5, MTRNR2L8, TMEM86A, PCED1B, ATF3, GSTO1, ACOT4, ADGRF1, BTNL8, S100A10, BUD31, SERPINB2, AKR1C3, RPL30, CYP4F12, TMEM212, MLPH, TNFAIP1, YBEY, BAMBI, CEBPA, SNHG6, STX4, MRPL52, C4BPB, SRSF9, MYRFL, NIPAL1, POMP, PLBD1, OR7E37P, THRB, SBDSP1, SEC61G, RRAGB, LINC00504, CALR, NDUFS4, COX6C, PSMA5, ARL16, LLPH, F2RL1, ZNF585A, MRPS28, AGR2, ABHD11-AS1, STARD10, S100A4, TOP1, TP53INP2, TRIM31, MGST3, EIF4ENIF1, NDUFS6, C8orf59, ATP5E, LINC01207, TMCC3, PLAC8, RPS25, DSEL, ALKBH3, YAE1D1, ATP5H, RPS21, LINC01559, TSPAN1, SLC7A7, LMTK3, GRHL1, SELK, TRIM16, MT-RNR1, GCNT3, TICAM1, SERF2-C15ORF63, MIR22HG, VNN1, GADD45B, FRAT2, ANKS4B, BMP2, DAD1, RPL36A-HNRNPH2, UFD1L, LMO7, LOC729348, SULT1C2, TRIP4, KRT14, TATDN1, TEKT4P2, IL1RN, ZNF738, RPL27, RPL14, KLF4, OCIAD2, TRIM10, PSMB10, ANXA2, INO80E, TMEM126A, EPHB6, GRAMD4, PLA2G10, AK6, SHD, MKNK1, TPRKB, MROH6, EHD1, TOMM40L, AKAP7, LOC389332, MUC2, ZCCHC9, TOPORS-AS1, BCCIP, ZFAS1, METTL3, CDKN2B, COX7A2, SFT2D2, LOXL4, SPECC1L, ENTPD5, BLNK, PLPP1, MT-RNR2, TRAPPC2L, PDIA6, NDUFA7, GGT6, GSAP, MT-TF, DUSP10, ARRDC4, PDCD10, PRDX1, IL18, C2orf72, EPB41L1, TFPI, CTSV, PYCARD, PAQR5, RAB27B, ARL14, SULT2B1, SFT2D1, KRT20, MCC, KCNQ1OT1, DUSP1, FUNDC2, PLA2G16, UQCR10, ITGA2, COX5B, S100A16, FOXD1, MBOAT1, ZNF706, KLF7, COMMD6, SKIL, SYT13, SF3B6, CEACAM5, SPATS2L, HIVEP1, LAMTOR5, CALML4, ZSWIM6, DNAJC7, SLC22A18AS, SNHG5, CXCL8, UEVLD, C14orf105, RHOC, CTC1, LEO1, PDGFA, EMG1, OTUD6B-AS1, ZNHIT1, RPL22L1, ONECUT2, NDUFS5, EMP1, ACYP2, ANXA4, NOP16, NDUFB8, MTERF3, MINOS1, RPL39, TPD52, RP9, TMEM171, PIGT, SGK2, LOC100131257, POLR2K, NDUFB9, VPS33B, GRHL2, EPS8L1, FOS, MTMR11, IER5, ZNF480, LRP8, GTF3C6, NDUFV2, M6PR, CLCN2, BOLA3, FUNDC1, DRAP1, TNFAIP2, MMP28, PHF14, SPINT2, ID3, C9orf85, SH3BGRL3, UGDH, CISD3, HEBP2, NMNAT1, NDUFA13, STYK1, PDSS1, EEA1, UGT2B15, SLC37A1, NDUFA4, S100A13, CLU, SURF2, ME1, SRGAP1, RABEPK, CAPG, SPECC1, BEND7, RPL7, MIR194-2HG, RIPK4, FDPS, GRAMD1B, CGN, POLD4, BMS1P20, PPP1R14D, RPL26, RPL36, CFTR, POLR1C, OVOL1, AGA, CADPS, C12orf57, RPL38, TACSTD2, REPS2, PNMA1, NDUFB2, CMC1, LSM6, HMGN5, RASEF, CASP4, CDC26, ADAMTSL5, MRPS17, MRPL47, GPKOW, GOLGA6C, RBM3, BCL2L15, DBI, SNX7, CUTC, BHLHE40, SEL1L, MALAT1, CDC42EP2, DUSP12, TP53I3, DDX23, C3orf14, UCHL3, SNRPE, ECI1, IFT20, ARAP2, MGLL, ZFAND2A, TLCD2, GADD45A, ID1, TRIM36, C12orf45, FNBP4, BPGM, GTPBP8, FIG4, SMIM19, SWAP70, LSM5, AATK, INPP1, COX7B, GPATCH11, PSME2, TM4SF4, MKKS, NR1I2, AES, TXNRD1, CTSA, SLC41A2, AHNAK, AKR1C1, TMEM144, ICT1, C5orf30, NAA10, ITSN1, DHRS3, RPS27L, PHF23, PTS, FA2H, RPL35A, KIN, RTCB, SARS, FRMD8, UBXN8, MLKL, TMEM208, MUS81, TIMM22, SNHG17, SNRPD2, TATDN2, ZPR1, COX7C, AIFM1, RPL34, SSU72, GTF2H5, TPD52L1, CRYZ, RPL29, FDX1, GSDMB, LHFPL2, RAB3GAP1, ISCA2, EPB41L4A, MGST2, MALL, ACSS2, NDUFA6, TOMM7, RPL17, PAWR, PRELID2, DCDC2, GSTP1, MCCC1, UST, MRPS15, PLEK2, TAGLN2, NR1H3, ARHGAP21, COX6A1, EIF3M, TIMM17A, CASP7, NDUFA9, TADA2B, ACBD6, KRT18, IRS2, TMEM54, EFNB2, BRMS1, TANK, TFB2M, EIF2B5, ASL, SLPI, RPS24, ATP5J, TCEB1, NDUFC1, DEDD2, FAU, DECR2, THAP6, NDUFAB1, ANKRD49, LOC729966, EIF3K, PFDN2, CTSS, HDDC2, DYNLL1, KIF13B, CARS, SQRDL, AHCYL2, HSD11B2, PLEKHA2, PSMA6, ITGB1BP1, FUT2, DNAJB11, NABP2, TXNDC9, ABL2, DIP2B, MRPL18 |
|  | Down | MYL9, PITPNC1, DAGLB, STK38L, PWWP2B, E2F3, ATF2, TERF2, PHF13, EPHB2, CNN2, KREMEN1, ERI1, TPRN, DIEXF, TOP3B, SLC25A51, MSX2, KPNA2, KIF22, TRERF1, DDX50, DMWD, CDC42EP3, FGFR1, DSN1, WWTR1, ATG4C, WHSC1, C4orf48, ABCA5, G6PD, ZNF703, PEX5, PIK3R2, UNK, PIR, CFAP36, PTAR1, SLC12A7, TOX, SNX33, P4HA1, IKBKG, ERI3, RANBP10, E2F4, NDE1, TMEM110, PGM2L1, ENC1, ATP6V1B2, ZFP3, MTMR10, NOX1, ZBTB33, ATP6AP2, PPDPF, PELP1, GCHFR, WDR45, TRIM41, SPATA13, ALDH1B1, PARP2, TCF12, MZF1, CDHR5, CLASP2, LYRM1, BANK1, CLDND1, HMG20B, LOC100506990, METRN, PPP2R4, CLDN2, PPIH, UGT2B17, PRKCDBP, PBX3, HADH, TRAF7, FKBP5, RNF2, CSK, BRMS1L, MTA1, SKA2, ZNF267, RRN3, ANP32E, AUTS2, RNF123, ATP6V0E2, NASP, RHNO1, GIGYF1, SEMA3B, PTPRA, H1FX, TCEA1, UBN2, KLHL42, C2orf88, TGFBI, ANKRD52, PPP1R1B, RMND5B, TXNDC16, FAF1, RCCD1, GPR153, C4orf46, FTO, PDP2, ICE2, PRSS23, KHSRP, DCP2, STAT1, SLC39A3, TMEM94, CDK2AP2, TWSG1, DBR1, SAC3D1, TTLL5, NLK, CHMP7, SP1, ZNF446, FKBP8, ANKRD29, STRN4, RNF26, TNS4, TMEM237, SAPCD2, SPNS2, PATZ1, ZNF264, PCK1, SPHK2, RBBP4, CPSF7, BMT2, SPIN3, C9orf16, REEP4, GGA2, TSHZ1, ZNF805, RFX7, TMEM173, MELK, MRE11A, SH3BP4, PSRC1, SESTD1, HSPA13, TCOF1, ROR1, GAS6, WDR89, CSNK2A2, RAB11B, LAMA5, EML3, INPP5K, ALDH7A1, LY6E, RCC1, ZNF12, CUL7, LARP1, ALKBH6, BNIP3L, HELLS, LRP6, FOXO3, MT1E, HLTF, SELO, TRAM2, PRR12, KDM4B, AURKA, FOXRED2, FHIT, SBNO2, MSH6, SOCS7, PRSS22, DEPDC1B, C16orf45, GAL3ST1, CDHR2, BRSK1, ITM2A, ZNF428, MDM4, CENPM, OXCT1, CDC23, SLC25A22, AP4S1, FNDC3A, TBC1D16, B4GALT6, ZNF510, MAD2L1, TADA3, TKFC, ZNF668, CD81, FBXO32, CENPN, SNX5, LRP5, LOXL1-AS1, LANCL1, LTBP4, TNKS, ADAT1, ERMARD, URB1, WDR90, FAM193B, HDAC7, PPP5C, CDCA7L, ZDHHC9, HILPDA, SLC12A2, MIS18BP1, SRBD1, FLVCR1, LMBRD1, GIMAP2, LOC107133515, ZNF571, ITFG2, TRIM47, PRKDC, TPGS1, BCL7A, POLD1, MT1F, CDK4, DCAF17, PPP1R35, CRTC1, ZRANB1, GPHN, HAUS6, UCK1, CCNT1, HHLA2, TMEM56, NCAPD3, FAM222A, MDFIC, RICTOR, NUAK2, POLE2, ITPR2, SLC48A1, ORAI3, THRA, CEP170, SLC25A37, FAM73B, TESC, RMI1, NDNF, HSD17B8, TBC1D5, PRRC2B, FAM234A, LINC01278, MIB2, CDKN2C, MAOB, RACGAP1, S100PBP, BCL11A, C19orf48, PALD1, SLAIN1, ABCB9, FEN1, TTK, CDADC1, NCAPD2, SMC4, MPV17L, CENPK, ZNF322, BBS1, ALDOC, CNPY3, CDCA7, SLC1A4, TULP3, TBC1D12, ZFYVE26, DTL, PNPLA2, CD3EAP, ADAT2, DNAJC27, LCORL, NOTCH3, SEZ6L2, C19orf66, CDK19, C16orf58, FAAH, CBX5, REN, SASH1, AAAS, ZKSCAN1, DYRK4, CDK16, PHTF2, LPCAT1, VWA1, IRF7, CDK1, SCG5, ANKRD9, NFIC, MYH10, SLC29A1, PRPS2, CNN3, DCBLD2, PRSS16, BCL2L12, TTI1, PRDM7, AKR1B1, GPX4, HMGA2, SIX5, SLC51A, CCDC137, ACVR1C, KANK1, RBL1, RNASEH2A, CCNB2, ZNF792, MED23, RGL1, GOLT1B, RRM1, ZDHHC1, LRSAM1, ANK3, AKT1, ZNF275, SOX9, TBC1D25, RBP2, NUCB1, ANAPC2, TM6SF2, EZH2, EDEM2, LRRC45, BRAT1, GATSL2, DENND5B, POT1, CCNA2, BARD1, RHPN1, IGIP, ZNF714, CDT1, HID1, SKI, FAM89B, KCNQ1, DCAF15, BEX3, CHDH, MT1G, BCAT2, VGLL1, GPX3, UBA7, NUDT16P1, LMNB2, LYZ, MCF2L, WNK2, PCM1, NEBL, PCYOX1L, CELF2, CENPBD1P1, LRFN3, PLK4, SP5, ZFP90, KIAA1324, FCF1P2, DIAPH3, TGIF2, MANBA, GSTM4, CENPU, RILPL2, MSLN, HIPK2, LRRN1, RPS6KA2, HSF2, SS18L1, RFC5, ZNF92, EIF2AK2, SUPT3H, POLE, PPME1, IGSF8, STMN1, ZNF22, FCHSD2, LOC100294145, ANGEL1, LRRC57, ZNF532, TGFBR3, CALCOCO1, QTRT1, IMPDH1, CDK2, DHTKD1, EFNA3, CNNM2, ZNRF3, CDC7, FAM8A1, SKA3, KIAA1549, NOTCH1, OAS3, LDLRAD3, MAD2L2, TBC1D17, MCM2, SNRNP70, SOX12, FAM81A, CDK6, CLSTN3, IQCB1, CCDC61, FGFR4, GLUL, ACADSB, ZNF445, PCMTD1, IFITM3, HJURP, NNT-AS1, CYB5R2, CENPF, PEG10, HSD3B7, CNOT7, HKR1, DEPDC1, GEN1, MIS18A, RGMB, ADRB2, STK36, PI4K2B, KAT2B, RPGRIP1L, CYP39A1, TK1, CHTF18, GRK6, FBXO5, SLC38A11, SRGAP2, MGME1, CCNF, THNSL1, TMEM170B, MCM4, SH3PXD2B, KBTBD7, TMCC1, KIF11, LFNG, GPR137B, ZBTB44, RAD51AP1, PCMTD2, ZNF367, ACCS, HEMK1, AMOTL1, MIR210HG, LOC100129550, PKD2, CHAF1A, GLCCI1, ADCK3, HIVEP2, MYLK, POLQ, TBC1D9, TECPR2, PAN2, EFNA4, VIM, CITED4, SLC51B, TRIM59, RNASEH1-AS1, C2CD4A, SNAPC1, RAB33B, BRIP1, SGK223, PDIA3, CNNM1, CRTAP, PKDCC, NCKIPSD, LIG1, NR1D2, OGFR, CDC20, ESPN, CENPH, ACE2, KCTD11, KIAA1586, TRIM28, GDF11, DBN1, TRAF5, FAM46C, TARBP1, RFC3, CDCA2, NMNAT3, RBMX, EFHC1, CENPO, ABCA2, UBXN7, SLC25A1, GLI4, CDCA5, POGLUT1, CKAP2, PDK2, DDX60, PRR26, DNA2, HDAC4, LRRC26, CASP2, PBK, CCSAP, EHBP1, BUB1, FIGNL1, MMS22L, CDKN2D, ZNF607, TP53I11, LINC01011, LRRC20, SORBS3, HELZ2, LOXL1, MAP4K2, BTBD2, SGSM2, TMEM175, LINC00261, GEMIN8, TAGLN, SLC40A1, UBASH3B, ABHD10, METTL4, TOP2A, MPP1, CEP85, ATAD5, ZNF282, RBM43, NDC80, PDZRN3, CHAF1B, KCNMB4, DDIAS, PTPRR, NRSN2, DMPK, ITPKB, RASSF5, XPNPEP2, SLC46A1, MPHOSPH9, PIGZ, TMEM201, BUB1B, GKAP1, EBLN2, NEMP2, IFT140, MANEA, PROX1, MIR99AHG, FAM120C, CCNB1, RGS12, KDELC1, EFCAB2, NOS1AP, TRIP13, SLC2A4RG, ABHD17A, WTIP, STXBP1, CDCA4, LOC643072, ZNF500, TAF1, PTGER2, EHMT2, KIF23, IFT80, IQGAP3, DDX58, SLC16A3, STX17, LGR5, FBXO44, MKI67, EPSTI1, CTU1, FRS3, DLGAP5, MELTF, KCNJ2, TNFRSF19, RHOBTB2, FAM111B, SLC25A35, KLHL24, CA8, LOC101927503, HMMR, SDC3, VAMP1, HDAC10, ORC6, MYBL1, ALG12, LRG1, GYLTL1B, RTKN2, ARVCF, TSPAN9, PRKCG, LOC100287497, RNF24, LOC100288152, SIX4, PRKCQ-AS1, TRMT61A, SLC11A2, CEP250, BEX4, CENPI, NUBPL, FRMD4A, CNFN, RECQL4, CENPE, SLC2A13, PRR11, ASPM, SH3TC2, TRIM62, HHLA3, BIRC5, E2F7, IFI27, CLSPN, OGFRL1, TPX2, ALDH6A1, YY2, KIF20B, CCDC146, LEMD1, BRCA2, AKNA, NOXO1, INAFM1, SLC6A8, CDC25C, IFI6, ZNF155, ZNF761, CTXN1, FOXM1, TDP1, DIXDC1, HOXC6, BACH1, CKB, SGO1, THRIL, MECP2, SNAPC2, SLCO4A1-AS1, SNTB1, SESN3, CDH11, AIF1L, NCAPH, RNF144A, SMO, CHMP1B2P, BHLHB9, AK7, MAP2K6, GRAMD1A, GPR63, TICRR, NINJ2, GAMT, SPAG4, ZNF771, MAP3K3, ARSB, LRTOMT, RAD54L, TCF19, ZC3H12C, FGF9, BNIP3, SGO2, MCM5, RRM2, CDK5R1, TACC3, EPHB3, SORBS1, HLA-DMB, DKFZP434I0714, C12orf66, ZNF204P, MCM10, ZNF385A, IDUA, TMEM132A, ANLN, THSD4, AHCTF1, ASF1B, ENO2, SP4, RBBP8NL, GPR155, AK9, SCMH1, BLACAT1, OBSL1, CADM1, CHST14, NXN, SKA1, ZSCAN2, SLC43A2, TROAP, ZNF311, LOC113230, UCP2, NUSAP1, ARHGAP23, SPC25, FN3K, PDLIM4, GMPR, CFAP69, ENPP3, NEURL1B, SAPCD1-AS1, C1orf112, CYP4X1, ASIC1, ZGRF1, DUSP19, SMTN, SERPINA1, CIT, PTK7, SUV39H1, CEACAM7, PRR36, MS4A10, XRCC3, H2AFX, ANXA6, SOD3, KIF18A, KLF11, SUSD2, ISYNA1, ZSCAN21, MUC6, LOC728743, HUNK, CYBRD1, ENPP5, MSI1, ACE, AQP5, CKAP2L, SLC38A4, PALM3, ADCY10P1, SLC39A4, CEP55, LMCD1, AURKB, ALDOB, IFITM1, NUP210, SFRP5, ZNF596, NRM, MME, HERC5, SLC23A2, ZNF772, SECTM1, E2F1, CACHD1, ANGPTL4, HS3ST5, C17orf78, SRGAP3, MX1, FAM189A2, SLC27A1, FAAP24, ASCL2, GBA3, CREB3L3, PODXL2, EMB, C1orf233, TMEM143, TGFB3, FUT1, MCM8, ZNF224, LYRM4-AS1, FGFRL1, ACRC, CCDC71, 44075, SLC26A9, COL6A1, FSCN1, GPR137C, PLK1, KMT5C, CENPQ, ZCCHC24, MX2, ZNF573, KIF20A, FREM2, CYB5RL, IFITM2, PRICKLE2, C10orf88, FADS6, WDR27, TRPA1, PLEKHG4, TCF4, KIFC1, SEC31B, RPSA, SBK1, ZNF230, NLGN2, LOC102724064, SYNPR, CHRM3, PHYHD1, MAGEE1, KIF15, GTSE1, LRP5L, MFHAS1, GBP4, USP2, MAP2, VTCN1, SLC25A27, C11orf84, CDKN2B-AS1, ZNF503, TIMP3, PRAF2, PRRX1, CA5B, KLK8, TOB1-AS1, PRDM16, SOX8, ESPL1, HES4, AHRR, RNF186, GTF2I, ZNF25, HBB, PPP2R2B, SMOC1, IFI44, ZNF653, FAIM2, CAMK1, PHLDB2, SCARA3, GLIS3-AS1, LMNB1, NRGN, GPR19, ZNF436-AS1, FAM86B3P, ANKRD1, PXDN, PXN-AS1, TUSC3, SLC6A4, OTOP3, NTF3, PTCHD4, KLHL34, INHBB, MFI2-AS1, HS3ST2, ZNF611, REEP1, GABRB2, ASRGL1, DFNB31, SNORA25, LOC105374952, KIF3C, MGAM, CD36, CXCR4, GAPLINC, LINC01489, HCG8, C1QTNF6, NREP, LOC100506127, IFI44L, DHRS2, HIP1, PRICKLE1, ARHGAP19, ZEB1-AS1, MYBL2, CCDC15, ZNF595, LOC644172, TNFAIP8L1, COL18A1, RIBC2, SOCS2, FMOD, LOC105376575, POM121, GLDC, RASAL2-AS1, ARNT2, APOBEC3H, HDX, EPOR, PCP4L1, MUC5AC, LNP1, ADH4, EPM2A, OSMR, RPS15AP10, DMD, LAMA1, MEP1B, GNAZ, LOC148709, ST3GAL4-AS1, PLEKHB1, TRPV4, EVL, ZNF502, ADGRL3, MTTP, UFSP1, LOC101559451, PKHD1, MPL, ATP6AP1L, FANCG, MACROD2, MIXL1, SLC7A9, MT1H, NEU4, C5orf38, FAM229A, OAS2, TEAD2, ANPEP, MEX3A, SLC39A8, TMIGD1, CRYBB2, PRRT1, CMPK2, TPTE2P5, ITGA5, AGER, PDE4A, SLC6A19, APOA4, SLC24A4, NOS2, ZNF853, AXL, ZBTB10, FABP6, TTC7B, ZBED3, EFR3B, LOC728175, LYPD8, LINC00959, SULT2A1, ZNF736, TMEM211, ZSCAN20, NXPH4, MAMDC4, CYP3A4, FBXL16, RSAD2, APOA1, LOC101928886, KIF24, NES, FOXJ1, NAT8, SMOC2, SLC17A8, KLK5, SLC5A11, G6PC, CYP17A1, KLK4, ABCA1, HEG1, DHDH, LINC00173, TSHZ2, HSD3B1, ZNF454, TMCC1-AS1, DUOXA2, DNAH7, APOB, EPB41L3, C11orf53, CPO, LOC101927178, TMEM198B, CHST13, MIR7111, DOK7, LOC101928403, CLIP3, LINC00605, PNCK, STXBP5-AS1, LRRC37A8P, APOC3, SLC7A2, ANKRD24, USP27X, SMLR1, LOC401261, KTN1-AS1, WIF1, LRRC37A4P, FGF14-AS2, TMEM254-AS1, DACT2, TMPRSS5, GLP1R, MORF4L2-AS1, AMH, LOC100240734, SHISA2, TMEM140, BEX1, ANTXR1, SH2B2, NUP210L, KRBA1, ZNF341-AS1, LOC100505549, B4GALNT4, BDNF, KIAA0825, PCP4, ZNF43, NOS3, CEACAM20, SLC19A3, C9orf24, MIR4697HG, LOC101926913, NOTCH4, LINC01612, PEAR1, DOC2A, APOBEC3G, SLC2A3, SLC28A1, ZNF709, ST6GALNAC3, PPP1R3G, KRTAP2-3, RIMS2, LRFN1, PLIN1, DRD4, GPR146, TRIM40, NDRG4, KLHL32, TOLLIP-AS1, FGD1, TP73, PTPRM, GATA4, LHB, LHFP, FZD2, BTN1A1, PDE3A, TRPM5, ENPEP, NEFL, CAPN6 |

| 60h | DEGs | Gene terms |
| --- | --- | --- |
|  | Up | BMP3, CHAD, KRT6A, PLAT, COL4A5, CXCL17, LOC283177, ALOXE3, CLCA1, KRT4, TTLL6, C6orf99, CHGA, KRT5, CD177, REP15, FCAMR, SI, LGALS2, DUOX1, CXCL14, SOCS1, UGT2A3, DMBT1, SLC2A5, IL18BP, TTR, BST1, MGC32805, TMEM74B, PRSS1, EHD2, MS4A8, C6, MFNG, SPINK5, POU2F2, GSTA2, ADH1C, FREM1, WFDC2, DPEP1, LOC100506688, S100A2, PFKFB1, GSTA1, PACRG, SULT2A1, C20orf24, NEB, CRYBG3, POU2F3, LUM, NR0B2, LOC101927356, C11orf98, LINC00506, SST, PRR19, SH3BP5-AS1, LOC100288911, TMEM229A, LPIN3, SLC2A2, CCR6, XDH, KRT13, ANKRD34A, VNN1, SLC52A1, L3MBTL4-AS1, CHST2, ZNF839, MYO18A, ABCC2, CCDC17, RHOF, ARC, MT1H, ACOX2, TNNC2, ZG16, POLN, CYP2B7P, LOC105372751, HOXA13, TMEM236, AFP, ENPP7P13, MT1G, PLA2G7, SIRPA, SPINK1, ZIM2-AS1, SLC26A11, CCDC30, GCG, MMP7, CDKN2B-AS1, UBXN10, ADTRP, BTNL9, LINC00670, BTNL3, PTPDC1, LYPD6B, CA1, MALRD1, NAT8, LEF1, NRCAM, LINC00550, SERPINA3, SEMA6A, SULT1A2, SLC5A9, ENTPD8, COLCA1, AQP7P1, CRYGS, CPO, RBP2, ZNF213-AS1, SLC17A4, CPS1, CLCF1, LOC729987, CDK20, ANXA13, LOC101929415, G0S2, HOXA1, AMDHD1, PCF11-AS1, FUOM, LINC00504, MALAT1, GBA3, PRSS2, CA3, SLC5A11, GPR85, NME2, DISC1-IT1, UBD, MT-TT, SLC7A7, CCDC144B, BLNK, DHDH, OLFM2, LONRF2, SLC7A8, SEPP1, APOD, PADI2, MINK1, TMEM253, ZNF593, HLA-H, LINC00842, OR7E91P, MARVELD2, FCGBP, BLOC1S1-RDH5, SNORD104, MUC3A, FAM155A-IT1, TGM2, KRT15, FLJ32255, CTSA, LOC729348, SLC38A5, LOC102724699, LOC101928103, ZNF471, MRAP2, HBP1, RBP4, LINC01602, PPIB, CTSO, ALOX15B, LOC100131257, ABCC6P1, AIMP2, MAOA, RPS26, ACPP, VSTM2L, PDZK1IP1, CAPN8, ZNF623, RPS10-NUDT3, PI3, MTG2, ADARB1, GPD1, ROR1-AS1, ANO1, EPB41L3, PRDM15, KIAA0226L, LINC01504, C15orf48, ICAM1, BTNL8, FAM118A, ACHE, PDXDC2P, LOC101927884, AADAC, PRLR, ONECUT2, DOK3, TXK, SNORA61, SPON1, EMC6, TIMP1, HFE, MT-TY, FCHSD1, GOLGA7B, RRN3P1, SLC43A1, LEAP2, CDK11B, CCL2, CYP4F12, AP2A1, ARRDC4, LINC01224, CACNA1D, ERICH4, CTNNA3, AGAP1-IT1, ACE2, CKMT1A, INPP5D, KIFC2, GSDMB, EGFL7, PIGR, KCNQ1OT1, ANO9, LOC101929486, SULT1E1, LOC101928936, PRELID1, TMEM212, PPP1R14D, NRP2, AKR1C2, CHN2, SLC5A1, SLC39A5, NME7, SULT1C2, SCAMP5, C1QTNF6, SUSD2, ASS1, CASP16P, STX4, STC2, PCCA, UVRAG, LOC101926898, CCND2, ZNF383, UG0898H09, RGS2, MOGAT2, VSIG1, SLPI, AKR1B10, CMTM3, KLHL13, HIST1H1C, ATP5J2-PTCD1, RARRES1, STX19, VAMP5, ABHD11-AS1, FAM47E, C16orf95, GCNT2, ARHGEF11, SLC37A4, S100P, CARS, ENDOG, LY6G5C, PLCG2, UGDH-AS1, FAHD2A, KHK, GDPD3, C2orf82, DSEL, SMPD2, M6PR, MKRN2OS, DDX23, FBXW8, CYP4F3, STARD10, EPHX2, DCBLD1, F5, GLOD5, C1orf198, PCK2, ATOX1, MUC2, FAXDC2, GSAP, SLC26A3, PGM1, ACOT8, IRAK2, PLA2G12B, LINC00483, CCL28, SPG20, TRIM10, KCNJ3, COA7, YTHDF3-AS1, SLC25A25-AS1, GOLT1A, PLA2G10, SOX9-AS1, IKBKE, CLCN5, GSTO1, NFKB1, PIGT, PSMB7, LOC102724908, PLBD1, SLC51B, CTSL, CARD6, GHET1, ZNF490, PRSS3, NRP1, NTMT1, NHS, CYP3A4, RN7SK, CTC1, MT1F, HAAO, TRIM29, DYRK1B, OR7E14P, PFDN6, LINC00659, NR1H3, C19orf70, FAM83F, MOCOS, STUB1, FTL, RAB26, FAM131B, MMP14, SGK2, TSPAN1, SDSL, NMB, RRP8, URM1, PBLD, KIAA0754, LOC101926889, LCN2, CRADD, STOM, AKAP7, BARX2, CYP3A5, ARPC3, KRT14, PDIA6, CA2, DDT, PID1, CD68, ZNF738, MKNK1, KLF7, FAM198B, BCL2L15, TSPAN7, ZNF865, MRPL11, CYP2C18, AKR1C4, BUD31, NR1H4, TACO1, CALR, HIF1A, TTC31, TGIF2-C20orf24, SERF2-C15ORF63, CYP2E1, FGFR3, SLC35G1, APOBEC1, NEIL1, CDKN2B, RAB3B, SPCS1, RASGEF1B, LAMB3, POLR3D, SLC3A1, CES2, TMEM183B, COX6C, B3GNT8, C14orf105, GFER, TMEM265, NDUFA13, PLPP1, RPS14P3, TIGD1, BMS1P20, NVL, BAAT, C19orf52, LOC729966, LGALS4, PSMC3, TFPI, ACOT4, SPNS3, HERC2P7, PRKACB, NPTN-IT1, TMEM144, MATN2, HTR1D, RHOC, ARRDC1-AS1, PDCD11, MYO7B, ASL, EIF3K, DPCR1, DYNLRB1, ATP7B, CRNDE, MUC1, INO80D, LGALS1, TLCD2, MT-TF, SMCR8, MTRF1, PRDM5, UPP1, LOC100129434, RNASE6, CBR1, HLA-DMA, MIR194-2HG, HTRA2, NACA, SOX6, NEK11, FABP2, TMEM219, LINC01003, ANO10, ENTPD5, ALDOB, MIR17HG, SMIM6, THBS3, ASTN2, CDX1, NDUFB9, METTL7B, AKR7A3, RERE, TKFC, DPP4, SH3RF2, MRPL32, MAB21L3, TTC39A, GTF3C6, TSIX, HMGN5, SLC25A20, HIST1H4C, CAPG, BLVRB, LAMC2, DST, VGLL4, IDNK, SULT1A1, CRIP1, DDC, KANSL1, ACKR4, P2RX4, PEX12, OSR2, SMIM24, NR5A2, IL1R2, ANKS4B, FUT6, ATG9A, BRI3BP, GOLGA2P5, SIL1, COLCA2, MAP2K5, NDUFS6, UQCR10, PEPD, HNF4G, MRPL52, CLDN15, THYN1, NTPCR, LOC101928118, ING4, CIB2, SEC14L2, PHF23, ADGRG7, BCL2L14, MLXIPL, LAMA3, SFMBT1, SLC46A3, PIK3CB, KRT19, HLA-F, C6orf106, PSME2, MYO15B, MYO1A, DAB2, CHMP2A, GRINA, QPRT, ST6GALNAC1, SLC27A3, OGDH, COX16, ZMAT2, MBOAT1, BCAS1, STAU2, BMS1P5, AREG, IFT20, LMTK3, XAF1, C5orf30, MAPKAPK5-AS1, SLC31A2, LOC101928820, PHLPP2, ZNHIT2, MDK, PMS2, CCDC93, STXBP4, MRPL23, HSPB1, PRAP1, XPNPEP1, CLCN2, GSS, ILKAP, GOLIM4, KIF13B, UROD, SCRN2, LRRC19, MCF2L-AS1, RGS14, MVB12A, ETFB, HADHA, AKR1A1, PDGFA, LMO4, ATP2C2, S100A4, SCAPER, EXOC6B, XYLT1, SLC52A3, LOC100420587, ATP5E, NDUFB8, NOC4L, BLCAP, CFB, LACE1, MTTP, LOC55338, RPS18, FDPS, KCNK6, NABP2, TAPBP, PRUNE, LINC01207, BDKRB2, GSTP1, CAMKK2, TRAPPC2L, WFS1, TRIM31, SORT1, SLIRP, C1orf115, AAMDC, DHRS7, PPFIBP2, COQ4, HUWE1, TPD52L1, SARS, POLD4, SLC41A2, SNX24, ZNF652, PSMB10, NDUFV2, TMEM220, GGT6, SYT13, MRPL27, MRPS17, NUDT1, TOMM40L, POP1, MGST3, SULT1C3, ALKBH2, VIPAS39, CALML4, SUCLG1, SHFM1, DEDD, PARP12, NUB1, NAA10, ADAMTSL5, SMG7, FRAT2, ATP9A, COG1, NIPAL3, STIP1, MFAP1, UPF3B, GATB, BEND7, TMEM138, CYP2J2, C2orf72, YDJC, GAB2, DALRD3, MRPL28, ALKBH3, THEM4, SIAE, HBEGF, PNP, PPIE, AGPAT2, MEP1A, ZFAND2A, METTL7A, PLD1, ABCF1, SMIM22, RPL36A-HNRNPH2, GALM, PRDX1, RNF213, FOXJ2, TP53TG1, GALNT5, ZFAND2B, BCAP31, PAQR8, STAP2, DGCR8, ZNHIT1, TRNAU1AP, MT1X, MAST2, SSU72, LUZP1, COX6A1, SAP30BP, DECR2, COMTD1, DHDDS, EBAG9, MRPS18C, DGAT1, PDE4DIP, ECM1, ALG14, AKR1C3, C18orf8, ADIRF, ZSWIM6, MUL1, C19orf33, NDUFA9, TARDBP, GDA, WDR46, HOPX, TRIM26, BDP1, APOBEC3F, FAM3A, SAT2, TP53I3, PLCB3, DENND2D, PLAGL2, SLC27A4, GCNT3, SPR, UTP23, SELK, CLYBL, ELL3, DCTN5, GLTPD2, NPC2, TP53INP2, ZMYM3, C1orf123, LAMTOR4, PPP1R12B, OTUD7B, SLC5A6, PARP6, PAPSS2, SIPA1L2, PDSS1, CISD3, SEC31A, AGR3, NUDT16, SEL1L, HCG18, ILVBL, METTL23, LRRC27, UGT2B15, MTMR11, ALDH1A1, STEAP3, USP28, MLX, CREBBP, TAPT1, GGA3, TM4SF5, NDUFB10, LINC01133, ATP1A1, TNFAIP2, MRPL20, IFT43, RMDN3, IDH2, TIMM22, MALL, SMUG1, SLC25A11, JADE2, RABEPK, QRSL1, SLC15A1, SLC37A1, CYSTM1, BET1L, ADCK1, DNAJB11, NEAT1, HYKK, NMRK1, PLA2G16, TXNL4B, ZNF444, LRRC16A, HGD, C14orf1, AP4B1, TTLL4, PRDX5, SIPA1L3, PRELID2, IL1RN, BHLHE41, NDUFS3, BRMS1, WDR60, DHRS3, MLPH, CDKN2AIPNL, CISD1, YIPF1, USMG5, AGMAT, ZNF346, GPATCH4, BTD, EPB41L4A, NDUFA11, BPHL, S100A6, CRYL1, RNF139, GADD45GIP1, HACL1, IVD, GABARAP, AIFM1, PRR13, ENOSF1, MKKS, COX5B, RIT1, UQCRC1, PAG1, MPDU1, TMEM208, ZZEF1, DDX55, ELOVL1, CMBL, VPS25, NDUFB7, THRB, PDCD6IP, MINOS1, ECHS1, NSRP1, EMG1, PCF11, OSBPL5, RPL14, RPP25L, MRPS36, EDF1, PGPEP1, SLC45A3, AARS, PANX1, KIAA1671, GPR137, TXNDC17, VPS18, TMUB2, LINC00342, RTCB, CASP4, ATP5H, SPRYD3, POP7, OGG1, NDUFAF5 |
|  | Down | PPP1R26, KIAA0319L, ZBTB7B, PICALM, SERPINA1, HHLA2, SNX5, NUBPL, SLC25A22, CD46, PTPN12, RAPH1, FAM135A, JUNB, TMEM106B, SPRTN, MYADM, PHF6, RNF216, BCL11A, POLR2F, CRAMP1, SPIN4, SGSM2, ZNF703, CD81, SCG5, CPSF7, LANCL1, SLC39A4, HIPK2, HNRNPD, LARP1, SRBD1, TAF1, MPZL2, LMNA, NRGN, NCKAP1, CPT1A, TRUB1, MTA1, RRP15, ISY1, CAAP1, CENPBD1P1, RRM2, TMEM184C, DCP2, FOXJ3, MTMR1, ZKSCAN1, BICC1, NFIC, SHANK2, SIN3B, SDF2L1, FRMD3, SRRT, KLHL15, SLC39A7, ID3, CLSTN3, AMIGO2, BBS2, COPS6, VGLL1, IGSF8, TMCC1, KLHL36, RAD51AP1, CAPZA1, KLHL21, CLDND1, H1FX, IMPDH1, STRN4, CRIM1, FMNL2, KREMEN1, TOR1AIP1, CCAR2, UBASH3B, ABL1, SDE2, SERINC5, TCN1, WDR89, PMS1, AMOTL1, DTD2, MTMR9, RAVER1, NFKB2, EPDR1, RMI2, PPP1R35, HID1, NAP1L4, POGLUT1, CORO1B, ALKBH6, 43897, FHOD1, GPX4, SLC25A40, C12orf29, TCFL5, CTXN1, ATP13A2, CASZ1, DYRK4, DMTN, MECP2, ZW10, CACTIN, MED16, TOR3A, TTK, TDP1, SFXN4, SDAD1, LINC00673, REEP4, CSE1L, ASAH1, KIAA1804, BZW1, VPS50, MCM5, TNFAIP8L1, COPS2, ATG4C, HSP90B1, SNX14, PHTF2, EXTL2, TMEM30A, SOX9, MTMR10, TAF2, BNIP3, RACGAP1, PPARD, SLC12A2, C6orf223, NSMCE3, ABHD12, ARMC6, RASA2, SLC38A2, ENTPD4, ARL6IP6, LOC100294145, SNAPC2, ITM2A, TNRC18, WWTR1, FBXO30, CENPU, POLE, LRP5, API5, TMTC3, ANO8, MIS18A, MINPP1, IFFO2, GYLTL1B, ADCK3, HERC2, THNSL1, EPHB2, TNKS, ZNF510, PCM1, RDX, DHTKD1, NOP14-AS1, ATF2, LONRF1, SLAIN1, PBX1, EMP1, DUSP8, LRRC20, MND1, SNX18, H2AFX, SND1, GDF15, IFT140, ANAPC2, ZNF805, SBF1, TRIM2, CHMP7, AKT1, NRF1, MIB2, RGMB, SERPINB9, SETDB1, CDC23, CEP85, GPR180, HOXB9, CNN2, HR, NUAK2, EFNA1, ZBED3, SOCS4, SIVA1, CHRNA5, CCNA2, C1orf233, KIF20A, NRSN2, TRAPPC6A, H6PD, TINAGL1, IDUA, RPUSD1, BMP4, RNF2, LTBP4, E2F3, GTSE1, RCBTB1, FGFR4, GPX3, MMP1, IMPAD1, CDK6, RILPL2, EMD, CBFB, TGFBR3, PTAR1, EIF4A2, TUBG1, ZFPM1, PDGFB, GAL3ST1, TBC1D17, PLA2G15, AIP, PLA2G4A, NUP214, SGPP1, CTGF, ZBTB33, ARFIP1, PCMTD1, ZXDB, NAT1, ABHD17A, PRKAG1, ARHGEF26, CKAP2, KANK1, PHLDA1, TRIM62, THAP12, FAM220A, SKI, FKBP2, SBNO2, PKD2, MSL2, C2orf54, ADM, PRKAA2, MOSPD3, CRTC1, SLC29A1, SH3BP4, SPEF2, NOTCH1, BIVM, ERICH2, MSMO1, SPIN3, INPPL1, RHOBTB3, STX17, TADA3, LCA5, AXIN2, PGAM5, FBXW2, TKT, ALDH6A1, SLC40A1, CDC14A, TSGA10, CFL2, NOS2, HELZ2, PRMT1, TCEA1, TTI1, ZDHHC1, ZNF22, EEPD1, PRAF2, ZNF710, RECQL4, TMEM56, KBTBD7, CA12, UBA2, ALDH4A1, BNIP3L, CLASP2, GLI4, EPHB3, ADRA2C, NXT2, RBMX, SP5, PKDCC, LOC113230, TRIM47, ZNF267, NXN, MMS22L, CDCA4, RFC3, ERP29, RAB33B, PNPLA2, ZNF792, ABHD8, GAS6, UNC5B, CEP72, SMARCA1, HNRNPLL, LMBRD1, FAIM2, NCDN, SESN1, PIM3, TOE1, CCNE2, KIF18A, CNN3, SLC6A8, ZNF264, FOXO3, MYL9, ZNF155, RNF186, MSX2, HYAL2, BAX, STAM2, BUB1B, UCP2, SPAG16, PPP3R1, SLC23A2, CNOT7, RPS6KA2, PRPS2, RBM38, ZNF771, ZNF649, MZF1, BEX3, ERCC6L, RNF138, LOC103344931, LYZ, HSPA13, PDLIM7, LIG1, SNRNP70, LRRN1, AQP5, EFCAB2, ROR1, TIPARP, AP4S1, MIR99AHG, C16orf45, TXNDC16, ANKRD9, ACTN1, GORAB, DOK4, LTN1, RAD51B, DACH1, SLC27A1, C16orf58, CTIF, NIFK-AS1, CCDC109B, BARD1, NOXO1, LRSAM1, TIFA, LOC100505666, LRRTM1, MYLK, BCYRN1, DEGS1, KATNBL1, ZNF282, HIVEP3, MIEF2, DFFB, TWSG1, TIMP2, SNRNP35, CRTAP, ZNF48, SNTB1, AAMP, DOK7, LAMA5, SLC25A1, CDK5R1, SMOC1, ARHGDIB, HOXC6, GLUL, FBXO5, KCNH2, AMN1, EXPH5, SEMA5A, PPP1R3B, TMED9, SYNPR, KDM4B, FKBPL, CYP4X1, ADH4, TP53I11, TMEM129, CCDC71, TUBB3, PRSS23, KRT7, LRFN3, REN, ZNF571, FMOD, UBXN7, TACC3, COL6A1, CDKN2C, KIF20B, COL18A1, PKP3, VTCN1, FSTL1, OAZ2, NUMA1, ALG12, PODXL2, PLEKHB1, SACS, TCEA2, ABHD10, MAP3K3, EHBP1, RALGDS, USP21, TEAD4, CPVL, KCNJ2, PRKCG, OLMALINC, TRIM28, RHOB, GPR161, KLK8, RCC1, 44075, LMNB1, FAM222A, FLNA, PXDN, TBC1D9, SLC11A2, ZCCHC24, MID2, MBNL2, SOD3, CCDC85B, SIX4, HDAC10, TARBP1, BANK1, SEMA3B, E2F1, BBS1, LPCAT2, LOC643072, CKB, GMPR, ZNF845, GEMIN8, RPL31P11, C12orf66, ZBED6CL, ERI1, ARHGEF10, COPG2, TPGS1, ERO1B, ZNF860, ASF1B, RBBP8NL, HIVEP2, METRN, VN1R2, ANLN, SPATA7, ENPP5, RNF166, TRMT61A, NEBL, AHCTF1, DDAH2, PHYHD1, ID4, GOLT1B, ATAD3A, ANXA6, PTPRR, PRKCDBP, ZNF607, CREB5, BTBD2, IRF2BP1, PLK1, SLC39A8, EML2, FGFRL1, SP4, LOC399815, SERPINB2, SOCS2, CLIC3, MFHAS1, KCTD11, LOC100506603, ZNF573, CITED4, CAV1, KATNAL1, GPR155, GYG2, GPATCH3, VWA1, CTSF, CEACAM7, ATF6B, DCBLD2, ZBED8, POF1B, RASSF1, IL1B, CHRM3, MDFIC, CADM1, ELOVL4, NOC2LP2, LMCD1, ZNF503, ZSCAN22, HS3ST2, ARMCX1, ARPC1B, ETFBKMT, ZNF416, LINC01106, BBC3, RASSF5, ZBTB42, ARHGAP23, DKK1, PDE10A, LOC729970, FAAP100, ARVCF, LOC101928238, TEAD2, ZC3H12C, HVCN1, BRAF, IFITM2, TMEM132A, MDFI, DAPL1, FBXL16, PELI3, CBX8, CD276, FXR2, SLC16A3, CHST14, STK38, IGFBP1, ZNF736, TCEANC, MAGEE1, SPACA6P-AS, ZNF28, ZNF225, PRSS22, HSPA1L, SMTN, SH3BP5L, SUZ12P1, KLF11, HUNK, ZFYVE21, TIMP3, LHB, C11orf84, CAMK1, NEU4, SOX8, MTBP, NR1D2, PRDM16, PDE3A, PTK7, TMEM136, KRTAP3-1, CXCR4, ZNF891, SOCS3, HES4, TRPA1, CYR61, LAMA1, KIFC1, TRPM5, PRRX1, VIM, EFR3B, HCG8, SLC26A9, DHRS2, PPARGC1A, ZNF468, PTGER2, PDIA3, DFNB31, FSCN1, SMOC2, FOXN3-AS1, PPP1R3C, FAM134C, SECTM1, MITF, LTBR, THRIL, LOC101926913, TAGLN, PLXNB3, GPR146, ITGA5, HBB, ABCA2, MEX3A, PIP5K1A, MUC5AC, C6orf15, GATA4, MIR4697HG, FZD2, HDX, INHBB, STX11, PKHD1, TNFRSF10A, KIF7, PDLIM4, KLK4, LOC100507437, RGS5, KLK5, PRORY, ZNF653, FAM46B, LOC101927402, SLC2A3, MFAP2, SH2B2, SLC29A4, LINC01612, NXPH4, RNF215, PGBD5, LOC101559451, RRAGD, ANKRD1, ZNF709, APOBEC3H, FLJ37201, GLP1R, NEFL, NRTN, ANTXR1, MORF4L2-AS1, ZNF43, ARNT2, LOC100268168, TRPV4, FOXJ1, SNED1, BMP8A, MAP2, ADGRL3, EPB41L4A-AS2, C1QL1, CARNS1, C1orf186, HCG17, CLDN6, KHDC1, LOC100240734, ST3GAL4, BEX1, NUP210L, KRBA1, SLIT3, ZNF583, LOC728175, ZNF433, NDP, CRYBB2, EMB, ST3GAL4-AS1, RIMS2, LIFR, TOB1-AS1, LHFP, CAPN6 |
