# Supplementary material for: Identification of Key Pathways and Genes in SARS-CoV-2 Infecting Human Intestines by Bioinformatics Analysis
Source: Biochem Genet. 2021 Nov 17;60(3):1076–94. doi: 10.1007/s10528-021-10144-w (PMC8596852; doi:10.1007/s10528-021-10144-w)
Supplement: Supplementary file 2 — Supplementary file2 (DOCX 17 kb) Table S2: Significantly enriched GO terms of DEGs [file 10528_2021_10144_MOESM2_ESM.docx]

| 24h |  |  |  |  |  |
| --- | --- | --- | --- | --- | --- |
| ID | GO:0140014 | GO:0000280 | GO:0000070 | GO:0010948 | GO:0007059 |
| Description | mitotic nuclear division | nuclear division | mitotic sister chromatid segregation | negative regulation of cell cycle process | chromosome segregation |
| pvalue | 8.25E-16 | 4.20E-13 | 7.94E-13 | 9.63E-13 | 4.91E-12 |
| geneID | RPL24/KIFC1/PLK1/ANLN/LRP5/BCCIP/TACC3/TPX2/KIF18A/HSPA1A/AURKB/CCNB1/AAAS/NUSAP1/MAD2L2/KIF11/CDC23/BIRC5/OBSL1/PHF23/CDC26/AURKA/SMC4/MYBL2/KIF20B/XRCC3/TRIP13/NCAPH/CUL7/MKI67/CENPE/GEN1/NCAPD2/CDCA5/ESPL1/CLASP2/RCC1/NCAPD3/DLGAP5/DUSP1/CEP85/CDC20/FBXO5/SGO1/TNKS/NDC80/BUB1B/RACGAP1/NAA10/CCSAP/DSN1/BUB1/CHMP7/KIF23/EREG/CENPF/MAD2L1/PSRC1/CENPK/KIF22/RGCC/CDC25C/PHF13/TTK/CDT1/NDE1 | RPL24/KIFC1/PLK1/ANLN/LRP5/BCCIP/TACC3/PDE3A/CALR/TPX2/KIF18A/HSPA1A/SGO2/AURKB/CCNB1/AAAS/NUSAP1/MAD2L2/KIF11/CDC23/BIRC5/OBSL1/PHF23/CDC26/AURKA/SMC4/RMI1/MYBL2/KIF20B/XRCC3/TRIP13/NCAPH/CUL7/MKI67/CENPE/GEN1/NCAPD2/CDCA5/ESPL1/CLASP2/RCC1/NCAPD3/FIGNL1/DLGAP5/DUSP1/CEP85/CDC20/FBXO5/SGO1/TNKS/MSX2/NDC80/BUB1B/RACGAP1/NAA10/CCSAP/MUS81/DSN1/BRIP1/RAD54L/BUB1/TOP2A/RAD51B/CHMP7/KIF23/EREG/CENPF/MAD2L1/ASPM/BRCA2/PSRC1/CENPK/KIF22/RGCC/MYBL1/CDC25C/PHF13/TTK/CDT1/PRDM7/NDE1 | KIFC1/PLK1/TACC3/KIF18A/AURKB/CCNB1/NUSAP1/MAD2L2/CDC23/PHF23/CDC26/SMC4/XRCC3/TRIP13/NCAPH/CENPE/GEN1/NCAPD2/CDCA5/ESPL1/NCAPD3/DLGAP5/DUSP1/CDC20/FBXO5/SGO1/TNKS/NDC80/BUB1B/RACGAP1/NAA10/DSN1/BUB1/CHMP7/KIF23/CENPF/MAD2L1/PSRC1/CENPK/KIF22/PHF13/TTK/CDT1 | EHMT2/PSMA5/CNOT7/E2F1/CDK6/PLK1/CBX5/ZNF385A/PRKDC/CDK4/CASP2/CALR/PSMB7/PKD2/PSMC3/PSMA6/RPS27L/AURKB/CCNB1/GTSE1/CDK2AP2/RRM2/EZH2/CLSPN/MAD2L2/RBBP4/RPL26/GADD45A/KLF4/AURKA/PSME2/XRCC3/TRIP13/TERF2/E2F4/OVOL1/GEN1/CDK2/ESPL1/CDKN2C/RNF2/PSMB10/DUSP1/CDC20/FBXO5/HMGA2/CRADD/TNKS/MDM4/NDC80/BUB1B/NAA10/MSH6/BUB1/CDC7/CCNF/CENPF/MAD2L1/CCL2/CDKN2B/E2F7/DTL/SUSD2/KAT2B/HMGN5/CDKN2D/RGCC/CDK1/CDC25C/TTK/TICRR/BRSK1/CDT1/RBL1 | KIFC1/PLK1/TOP1/TACC3/KIF18A/SGO2/AURKB/CCNB1/MIS18A/NUSAP1/MAD2L2/CDC23/CENPQ/BIRC5/PHF23/CDC26/SMC4/RMI1/XRCC3/TRIP13/NCAPH/MKI67/CENPE/SKA2/GEN1/NCAPD2/CDCA5/ESPL1/RCC1/NCAPD3/DLGAP5/DUSP1/CEP85/CDC20/FBXO5/SGO1/TNKS/NDC80/FEN1/SPC25/BUB1B/RACGAP1/NAA10/HJURP/MUS81/DSN1/BRIP1/SKA1/BUB1/TOP2A/CHMP7/KIF23/CENPF/MAD2L1/CENPN/CDCA2/PSRC1/CENPK/TOP3B/KIF22/CSNK2A2/BEX4/PHF13/TTK/SKA3/CDT1/NDE1 |
| Count | 66 | 81 | 43 | 74 | 67 |

| 60h |  |  |  |  |  |
| --- | --- | --- | --- | --- | --- |
| ID | GO:0044282 | GO:0006631 | GO:0001676 | GO:0071466 | GO:0033559 |
| Description | small molecule catabolic process | fatty acid metabolic process | long-chain fatty acid metabolic process | cellular response to xenobiotic stimulus | unsaturated fatty acid metabolic process |
| pvalue | 1.10E-08 | 4.67E-07 | 8.49E-07 | 2.26E-06 | 3.65E-06 |
| geneID | PGM1/ABHD10/GLUL/QPRT/AKT1/AKR1A1/HADHA/PNP/ETFB/CYP4F12/ADH4/DDAH2/PCK2/PCCA/AKR1B10/SULT2A1/ENTPD5/ENOSF1/LRP5/ECHS1/TKFC/ACOX2/HYAL2/OGDH/HGD/ACOT8/CRYL1/CPT1A/ALDH1A1/GALM/KHK/IVD/AKR1C3/PPARD/ALDH6A1/SULT1A2/APOBEC1/LPIN3/ENTPD4/NUDT16/IDNK/DECR2/ALDH4A1/ENTPD8/SDSL/ACOT4/HAAO/SULT1A1/NUDT1/IDUA/ADTRP/SULT1E1/FUT6/APOBEC3H/HACL1/SLC27A4/DHDH/UPP1/AMDHD1/ALDOB/CARNS1/APOBEC3F/CYP4F3/ETFBKMT/NOS2/HYKK/PLA2G15 | ALOXE3/NDUFS6/GSTP1/MSMO1/GSTA1/AKT1/PLA2G10/HADHA/ETFB/CYP4F12/NR1H3/CAV1/PCK2/DEGS1/CYP2C18/PCCA/GPX4/CBR1/ELOVL1/CES2/ECHS1/EPHX2/ACOX2/PRKAG1/PPARGC1A/ACOT8/CRYL1/CYP2J2/CPT1A/AKR1C2/AKR1C4/IVD/AKR1C3/BAAT/THEM4/PPARD/SLC27A1/LPIN3/SLC45A3/DECR2/ACOT4/MLXIPL/ADTRP/IL1B/HACL1/SLC27A4/ALOX15B/PRKAA2/CYP3A4/PLA2G4A/ELOVL4/CYP2E1/CYP4F3/ETFBKMT/SLC27A3/PLA2G15 | ALOXE3/GSTP1/GSTA1/PLA2G10/CYP4F12/CYP2C18/GPX4/CBR1/ELOVL1/EPHX2/ACOT8/CYP2J2/CPT1A/AKR1C3/SLC27A1/ACOT4/ADTRP/SLC27A4/ALOX15B/CYP3A4/PLA2G4A/CYP2E1/CYP4F3/SLC27A3 | GSTP1/GSTO1/GSTA1/ASS1/MGST3/ARNT2/PCK2/CYP2C18/DPEP1/GSTA2/E2F1/ABCC2/CES2/EPHX2/AIP/AADAC/PPARGC1A/CYP3A5/CYP2J2/AKR7A3/BAAT/CMBL/SULT1A2/PDGFB/BPHL/SULT1A1/NAT1/ICAM1/UGT2B15/CCNA2/CYP3A4/CYP2E1 | ALOXE3/GSTP1/GSTA1/PLA2G10/CYP4F12/DEGS1/CYP2C18/CBR1/ELOVL1/CES2/EPHX2/ACOT8/CYP2J2/AKR1C2/AKR1C4/AKR1C3/DECR2/IL1B/ALOX15B/PLA2G4A/ELOVL4/CYP2E1/CYP4F3 |
| Count | 67 | 56 | 24 | 32 | 23 |
