# Supplementary material for: Identification of Key Pathways and Genes in SARS-CoV-2 Infecting Human Intestines by Bioinformatics Analysis
Source: Biochem Genet. 2021 Nov 17;60(3):1076–94. doi: 10.1007/s10528-021-10144-w (PMC8596852; doi:10.1007/s10528-021-10144-w)
Supplement: Supplementary file 3 — Supplementary file3 (DOCX 16 kb) Table S3: Significantly enriched KEGG terms of DEGs [file 10528_2021_10144_MOESM3_ESM.docx]

| 24h |  |  |  |  |  |
| --- | --- | --- | --- | --- | --- |
| ID | hsa04110 | hsa05222 | hsa03030 | hsa04218 | hsa04932 |
| Description | Cell cycle | Small cell lung cancer | DNA replication | Cellular senescence | Non-alcoholic fatty liver disease |
| pvalue | 1.40E-10 | 1.25E-05 | 1.48E-05 | 6.09E-05 | 6.19E-05 |
| geneID | E2F1/CDK6/PLK1/MCM5/PRKDC/CDK4/CCND2/ORC6/CCNB1/MAD2L2/CDC23/GADD45A/CDC26/MCM4/E2F4/CDK2/ESPL1/CDKN2C/MCM2/CDC20/E2F3/ANAPC2/GADD45B/BUB1B/TGFB3/BUB1/CDC7/CCNB2/MAD2L1/CDKN2B/CDKN2D/CDK1/GADD45G/CDC25C/TTK/CCNA2/RBL1 | E2F1/LAMA3/CDK6/LAMC2/NOS2/CDK4/AKT1/COL4A5/TRAF5/ITGA2/GADD45A/LAMA5/PIK3R2/LAMA1/LAMB3/CDK2/IKBKG/FHIT/E2F3/GADD45B/COL4A1/CDKN2B/GADD45G | MCM5/POLE/RFC5/RFC3/MCM4/RNASEH2A/POLD4/LIG1/MCM2/FEN1/POLE2/DNA2/POLD1 | MAP2K6/E2F1/CDK6/GATA4/CALML4/CDK4/CCND2/AKT1/CCNB1/HIPK2/RBBP4/GADD45A/CXCL8/MYBL2/RASSF5/E2F4/PIK3R2/FOXM1/CDK2/ITPR2/TRPV4/E2F3/GADD45B/TGFB3/CCNB2/CDKN2B/FOXO3/CDK1/GADD45G/CCNA2/RBL1 | NDUFS6/NDUFB3/UQCR10/COX7A2/NDUFB9/COX5B/COX6C/NDUFB8/NDUFA13/NDUFA7/COX6A1/NDUFB2/NDUFS5/NDUFC1/AKT1/NDUFS4/NDUFA9/CASP7/NDUFA4/COX7B/NDUFV2/COX7C/NDUFA6/NDUFAB1/CXCL8/PIK3R2/CEBPA/IRS2/NR1H3/CYP2E1 |
| Count | 37 | 23 | 13 | 31 | 30 |

| 60h |  |  |  |  |  |
| --- | --- | --- | --- | --- | --- |
| ID | hsa05204 | hsa04978 | hsa04932 | hsa00983 | hsa00980 |
| Description | Chemical carcinogenesis | Mineral absorption | Non-alcoholic fatty liver disease | Drug metabolism - other enzymes | Metabolism of xenobiotics by cytochrome P450 |
| pvalue | 4.72E-05 | 9.55E-05 | 0.000140545 | 0.000304064 | 0.000587424 |
| geneID | GSTP1/GSTO1/GSTA1/MGST3/ADH4/CYP2C18/GSTA2/ADH1C/SULT2A1/CBR1/UGT2A3/CYP3A5/AKR1C2/SULT1A2/SULT1A1/NAT1/UGT2B15/CYP3A4/CYP2E1 | ATP7B/FTL/ATOX1/MT1G/ATP1A1/SLC5A1/SLC11A2/MT1H/MT1F/MT1X/SLC26A3/SLC40A1/SLC26A9/CLCN2/SLC39A4 | NDUFB9/NDUFA13/UQCR10/COX6C/NDUFS6/NDUFB8/NDUFV2/AKT1/COX6A1/NDUFB10/NDUFA11/NR1H3/BAX/COX5B/UQCRC1/NDUFA9/NDUFB7/PIK3CB/PRKAG1/NDUFS3/NFKB1/SOCS3/MLXIPL/IL1B/MLX/PRKAA2/CYP2E1 | GSTP1/GSTO1/GSTA1/XDH/MGST3/GSTA2/CES2/UGT2A3/NME2/NME7/NAT1/IMPDH1/UGT2B15/UPP1/CYP3A4/CYP2E1/RRM2 | GSTP1/GSTO1/GSTA1/MGST3/ADH4/GSTA2/ADH1C/SULT2A1/CBR1/UGT2A3/CYP3A5/AKR7A3/DHDH/UGT2B15/CYP3A4/CYP2E1 |
| Count | 19 | 15 | 27 | 17 | 16 |
